# Secondary thalamic neuroinflammation associates with disturbed corticothalamic connectivity in a model of severe traumatic brain injury in male rats—a longitudinal study

**DOI:** 10.1093/cercor/bhaf337

**Published:** 2026-01-09

**Authors:** Lenka Dvořáková, Raimo A Salo, Petteri Stenroos, Kimmo Jokivarsi, Jenni Kyyriäinen, Ekaterina Paasonen, Eppu Manninen, Mikko Kettunen, Pekka Poutiainen, Alejandra Sierra, Jaakko Paasonen, Olli Gröhn

**Affiliations:** A. I. Virtanen Institute for Molecular Sciences, University of Eastern Finland, Neulaniementie 2, FI-70211, Kuopio, Finland; A. I. Virtanen Institute for Molecular Sciences, University of Eastern Finland, Neulaniementie 2, FI-70211, Kuopio, Finland; A. I. Virtanen Institute for Molecular Sciences, University of Eastern Finland, Neulaniementie 2, FI-70211, Kuopio, Finland; A. I. Virtanen Institute for Molecular Sciences, University of Eastern Finland, Neulaniementie 2, FI-70211, Kuopio, Finland; A. I. Virtanen Institute for Molecular Sciences, University of Eastern Finland, Neulaniementie 2, FI-70211, Kuopio, Finland; A. I. Virtanen Institute for Molecular Sciences, University of Eastern Finland, Neulaniementie 2, FI-70211, Kuopio, Finland; Neurocenter, Kuopio University Hospital, Puijonlaaksontie 2, FI-70210, Kuopio, Finland; A. I. Virtanen Institute for Molecular Sciences, University of Eastern Finland, Neulaniementie 2, FI-70211, Kuopio, Finland; A. I. Virtanen Institute for Molecular Sciences, University of Eastern Finland, Neulaniementie 2, FI-70211, Kuopio, Finland; Diagnostic Imaging Center, Kuopio University Hospital, Puijonlaaksontie 2, FI-70210, Kuopio, Finland; A. I. Virtanen Institute for Molecular Sciences, University of Eastern Finland, Neulaniementie 2, FI-70211, Kuopio, Finland; A. I. Virtanen Institute for Molecular Sciences, University of Eastern Finland, Neulaniementie 2, FI-70211, Kuopio, Finland; A. I. Virtanen Institute for Molecular Sciences, University of Eastern Finland, Neulaniementie 2, FI-70211, Kuopio, Finland

**Keywords:** diffusion tensor imaging, functional connectivity, lateral fluid percussion injury, light sedation functional magnetic resonance imaging, positron emission tomography

## Abstract

Traumatic brain injury (TBI) is one of the leading causes of death and disability worldwide. The initial injury initiates a cascade of secondary injury mechanisms, including neuroinflammation and disruption of brain connectivity. In this study, we used the lateral fluid percussion injury model of TBI to investigate the relationship between secondary thalamic inflammation and corticothalamic connectivity disruptions. For this, we followed rats for six months post-injury, during which functional magnetic resonance imaging (fMRI) was conducted under light sedation, as well as diffusion tensor imaging (DTI) and positron emission tomography (PET). PET imaging with [^18^F]-FEPPA revealed neuroinflammation in the subacute stage in several ipsilateral thalamic nuclei, including the ventral posterior nucleus and lateral nucleus. In the fMRI analysis, we observed initial corticothalamic hypoconnectivity, which partially resolved by six months post-injury. DTI showed persistent increased mean, axial, and radial diffusivity in the ipsilateral thalamic nuclei from two months post-injury. Histological examination confirmed chronic thalamic neuroinflammation and neuronal loss eight months post-TBI. Correlation analyses showed that subacute thalamic neuroinflammation was associated with long-term structural and functional changes. These findings suggest that secondary thalamic inflammation contributes to enduring corticothalamic connectivity disruptions, which may underlie cognitive and sensorimotor deficits observed after TBI.

## Introduction

Traumatic brain injury (TBI) is a major global health issue and a known risk factor for various neurological disorders, including epilepsy, Alzheimer’s disease, and Parkinson’s disease (Brett et al. 2022). The initial injury, caused by an external mechanical force to the head, triggers a cascade of metabolic, cellular, and molecular events collectively referred to as secondary injury (Wang et al. 2014). In recent years, neuroinflammation has been recognized as a key part of secondary injury (Kuo et al. 2022).

Neuroinflammation is a complex inflammatory response of the central nervous system, with microglial activation being one of the central features. While the initial inflammatory response serves a protective function and promotes tissue repair, prolonged microglial activation can become detrimental (Donat et al. 2017). The inflammatory response begins at the primary site of the cortical injury immediately after the insult. Importantly, secondary neuroinflammation has been reported in brain regions remote from the initial injury site, such as the thalamus and hippocampus, which were not directly affected by the mechanical insult (Donat et al. 2017; Necula et al. 2022). Increased microglial activation in ipsilateral cortex, hippocampus, and thalamus has been reported in animal models up to one year post-injury (Loane et al. 2014). Clinical investigations using positron emission tomography (PET) have revealed increased binding of a neuroinflammation tracer in the ipsilateral thalamus, putamen, and internal capsule up to 17 years post-injury (Ramlackhansingh et al. 2011). This persistent inflammatory state was associated with vestibulomotor performance in preclinical studies (Dickerson et al. 2020), and chronically elevated serum inflammatory markers have been suggested to relate to cognitive impairment and negative outcomes in TBI patients (Kumar et al. 2015; Milleville et al. 2021; Collazos et al. 2025).

The thalamus serves as a central relay hub in the brain (Fama et al. 2015; Shepherd and Yamawaki 2021), making it a vulnerable node of circuit dysfunction following TBI (Holden et al. 2021). In a mouse model of TBI, the thalamic alterations contributed to corticothalamic circuit hypersynchrony, which led to sleep disruptions and spike activity (Holden et al. 2021). Furthermore, a preliminary feasibility study of deep brain stimulations targeting corticothalamic circuits reported improvements in executive control and behavioral and motor functions (Schiff et al. 2023). These findings underscore the importance of corticothalamic connectivity in the post-injury processes and the potential recovery pathways.

Functional magnetic resonance imaging (fMRI) and diffusion tensor imaging (DTI) provide non-invasive methods to study the corticothalamic connectivity alterations following TBI. Apart from assessing structural connectivity, DTI metrics can also provide information about microstructure, including clear associations with neuroinflammatory processes (Budde et al. 2011). In contrast, the impact of neuroinflammation on functional connectivity as measured by fMRI remains unclear. Thompson et al. found that acute levels of serum neuroinflammation biomarkers have correlated with functional hypoconnectivity measured in TBI patients in the subacute period (Thompson et al. 2015). Preclinical models complement these findings, as neuroinflammation in the ipsilateral thalamus correlated with its network strength at 7 days post-injury (Vinh To et al. 2022). However, TBI is a progressive disease, and even though the most dynamic changes occur in the acute and subacute phase (days to weeks post-injury), pathological evolution persists through the chronic phases (Yasmin et al. 2019). Therefore, longitudinal animal studies of connectivity are essential to fully understand the network disruptions and their implications for prognosis in clinical practice. Only a few preclinical studies that utilized imaging to study the brain connectivity have followed the animals past 1 month post-injury, and those did not include any assessment of neuroinflammation (Mohamed et al. 2021; Sharma et al. 2025). Therefore, the effect of inflammation on the chronic progression of corticothalamic connectivity remains unclear.

Based on the clinical and animal data, we formulated a hypothesis that secondary thalamic inflammation drives chronic corticothalamic circuit disruptions. To test this hypothesis, we investigated the long-term impact of TBI on corticothalamic connectivity and its interrelationship to secondary thalamic inflammation. To achieve this, we conducted longitudinal fMRI, DTI, and PET imaging over 6 months in rats following TBI. The detected thalamic neuroinflammation in the subacute stage correlated with chronic corticothalamic connectivity disruptions and structural changes. These imaging findings were further supported by histopathology analysis, which confirmed the presence of chronic neuroinflammation and neuronal loss in the ipsilateral thalamus at 8 months post-injury.

## Materials and methods

**Fig. 1:**
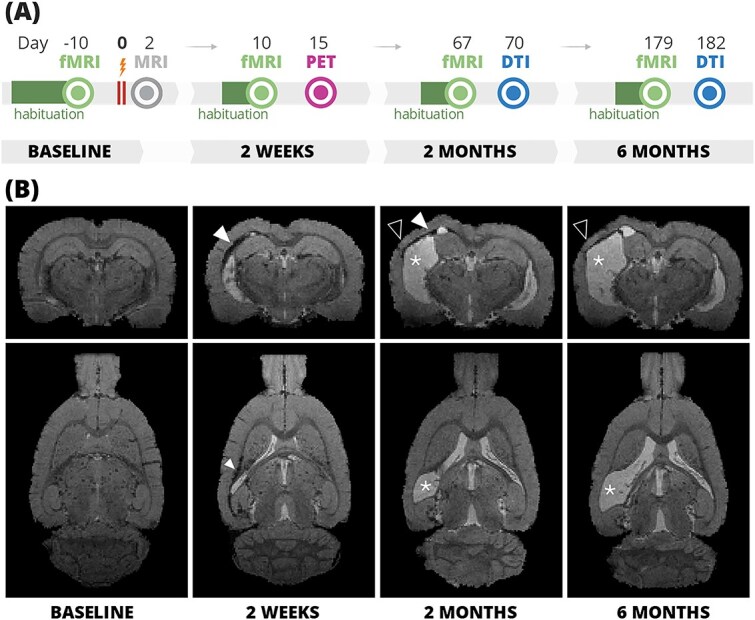
(A) Timeline of the experiments and (B) TBI lesion progression in a representative animal at the four timepoints: Baseline, 2 weeks, 2 months, and 6 months after the injury. Coronal and sagittal sections of T2^*^ weighted images that were acquired during the fMRI session. White arrowheads denote the signal hypointensity caused by microbleeds or iron residues, asterisks denote enlarged ipsilateral ventricles, and black arrowheads denote cortical thinning. DTI, diffusion tensor imaging; fMRI, functional magnetic resonance imaging; MRI, magnetic resonance imaging; PET, positron emission tomography. Partially created in https://www.BioRender.com.

The timeline of imaging experiments is shown in Fig.  1A. In the baseline (BL), fMRI was measured. Following the trauma, anatomical magnetic resonance imaging was performed at 2 days post-injury (D2) to confirm the injury location and severity. At 2 weeks (W2) post-injury, representing the subacute phase, animals underwent fMRI to assess changes in functional connectivity (FC) and PET imaging to evaluate neuroinflammation. Subsequent imaging sessions included fMRI and DTI sessions at 2 months (M2) and 6 months (M6) to explore chronic FC disruptions and structural changes.

### Animals and the model of traumatic brain injury

All animal procedures were approved by the Finnish Animal Experiment Board (license number R-ESAVI-2017-012704). Adult male Sprague Dawley rats (285 ± 34 g; Envigo Laboratories B.V., Horst, Netherlands) were used. A severe traumatic brain injury was induced by lateral fluid-percussion injury (LFPI) (Mishra et al. 2014) following the protocol described in Kharatishvili et al. 2006. Animals were anesthetized with a single i.p. injection (6 ml/kg) of a mixture containing sodium pentobarbital (58 mg/kg), chloral hydrate (60 mg/kg), magnesium sulfate (127.2 mg/kg), propylene glycol (42.8%), and absolute ethanol (11.6%), and a craniotomy was performed on the left convexity centered between bregma and lambda. A severe traumatic brain injury was induced with a fluid percussion injury device (Model FP 302, AmScien Instruments, Virginia, United States) with the force of the impact adjusted to a target value of 3 atm, resulting in a force of the impact of 2.6 to 3.3 atm for 59 animals. A control group of 14 animals was sham-operated (SHAM), where all procedures were otherwise the same, except that fluid percussion was omitted. Rats with abnormal findings (non-impact-related injuries or excessive subcranial hemorrhage in TBI animals or any cortical lesion in SHAM animals) were excluded from the study and sacrificed. Approximately 22% (13/59) of TBI and 7% (1/14) of SHAM animals died during or within a day after injury, and 9% (4/46) of TBI and 8% (1/13) of SHAM animals were excluded due to the findings at 2 days post-injury. A further 6 TBI and 1 SHAM animals were excluded or died at a later stage in the study due to various causes, including adverse reaction to anesthesia (4 TBI, 1 SHAM), development of subcutaneous tumor (1 TBI), or abnormal anatomical finding detected at a later imaging timepoint (suspected excess of subarachnoid CSF, 1 TBI). Hence, 47 animals (36 TBI, 11 SHAM) were included in the further study.

### Magnetic resonance imaging

All magnetic resonance imaging was performed with a 7-T/16-cm horizontal Bruker Pharmascan system (Bruker BioSpin, Ettlingen, Germany). An actively decoupled standard Bruker quadrature resonator volume transmit RF-coil and a quadrature surface receive coil pair optimized for rats were used. Anesthesia was induced with isoflurane in a mixture of 70% N_2_O, 30% O_2_. The breathing rate was followed during the imaging and was kept between 60 and 70 per minute by adjusting the isoflurane anesthesia level. The temperature of the animals was maintained at 37°C with heated water circulation in the animal bed.

For the anatomical imaging at D2, T2-weighted coronal multi-slice images were acquired with a fast spin echo (TurboRARE) sequence with TE = 40 ms, TR = 4000 ms, field of view 30 × 30 mm^2^, matrix size 256 × 256, RARE factor 8 (echo train length 8 echoes, echo spacing 13.3 ms), 2 averages. Additionally, T2^*^-weighted images were acquired with a three-dimensional multi-gradient echo sequence (MGE) with the following parameters: TR = 68 ms, TE = 2.73 ms, echo spacing 2.9 ms, echoes 13, flip angle 16°, field of view 25.60 × 19.52 × 12.80 mm^3^, matrix size 160 × 122 × 80, and voxel size 160 μm^3^.

#### Functional magnetic resonance imaging

For fMRI measurements, we used light sedation of 0.5% isoflurane with a body restraint approach described previously (Dvořáková et al. 2021). Altogether, 4 fMRI sessions were performed at BL, W2, M2, and M6. Before each measurement, the animals were habituated to reduce stress and motion during the measurement. The daily habituation time was gradually increased from 15 to 35 min over three days before the baseline measurement, and from 20 to 35 min over two days before each of the consequent measurements.

The fMRI was measured with a gradient-echo echo-planar imaging (EPI) sequence with the following parameters: TR = 1 s, TE = 18 ms, 1500 volumes, bandwidth = 200 kHz, 17 slices, slice thickness 1 mm, no gaps between slices, matrix size 64 × 64, field of view 3 × 3 cm^2^, and saturation slabs in four directions. Anatomical images were acquired with the same imaging sequence as in the anatomical imaging session.

#### Diffusion tensor imaging

DTI was collected at M2 and M6, 3 days after the respective fMRI timepoint. The imaging protocol used here was adapted from the EpiBiosS4Rx study (Sharma et al. 2025). Shortly, animals were anesthetized with isoflurane, and DTI was acquired with three-dimensional spin-echo EPI with TR = 1 s, TE = 26 ms, field of view 24.0 × 18.5 × 13.0 mm^3^, matrix size 96 × 74 × 52 (96 readout steps, 74 phase encoding steps, 52 2^nd^ phase encoding steps) with diffusion gradient duration of 4.2 ms, diffusion gradient separation of 12 ms, and b value = 2800 s/mm^2^ in 42 uniformly spaced diffusion directions and 4 non-diffusion-weighted images.

#### Positron emission tomography

A subgroup of animals (*n* = 34; out of which 25 TBI) underwent PET imaging using a translocator protein (TSPO) radioligand [^18^F]-FEPPA at W2 as described before (Yasmin et al. 2019). The measurement was performed on Inveon DPET scanner (Siemens Medical Solutions, Knoxville, TN, United States) under the same conditions detailed in Yasmin et al. Briefly, the animals were anesthetized with isoflurane, injected with [^18^F]-FEPPA (25.49 ± 3.14 MBq) via the tail vein, and imaged with a dynamic PET for 70 min. The structural reference images were taken immediately after the PET acquisition with a CT scanner (Flex X-O, Gamma Medica-Ideas, Northridge, CA, United States).

### Tissue preparation and histology

After the in vivo imaging, animals were implanted with 5 screw electroencephalography (EEG) electrodes in the skull under isoflurane anesthesia. At 8 months post-injury, after video-EEG recording to rule out the spontaneous epileptiform activity (data not included), all animals were perfused, and the brains were collected and prepared for histology. Histological analyses were performed on those animals that had a successful PET measurement to determine the inflammation status at the chronic stage. The brains were sectioned in the coronal plane (30 μm, 1-in-5 series) using a sliding microtome. The brain sections were stained with either Nissl or gold chloride to assess the cytoarchitecture and myeloarchitecture, respectively. To confirm the glial cell activation, a small representative group (3 TBI, 2 SHAM) was labeled with ionized calcium-binding adaptor molecule 1 (IBA1) for microglia, glial fibrillary acidic protein (GFAP) in astroglia, and 4′,6-diamidino-2-phenylindole (DAPI) for nuclei. The full protocol of the tissue preparation is described in detail in the Supplementary material.

## Data analysis

All data were processed and analyzed with in-house created scripts, Snakemake (https://snakemake.github.io/, Köster and Rahmann 2012), Python (version 3.10, https://www.python.org/downloads/), Advanced normalization tools (ANTs; http://stnava.github.io/ANTs/, Avants et al. 2009), FSL (version 6.0, https://fsl.fmrib.ox.ac.uk/fsl/fslwiki/), QuPath (version 0.5.1, Bankhead et al. 2017), and Quantitative Imaging Toolkit (QIT, https://cabeen.io/qitwiki, Cabeen et al. 2013), unless stated otherwise.

### Regions of interest selection

**Fig. 2:**
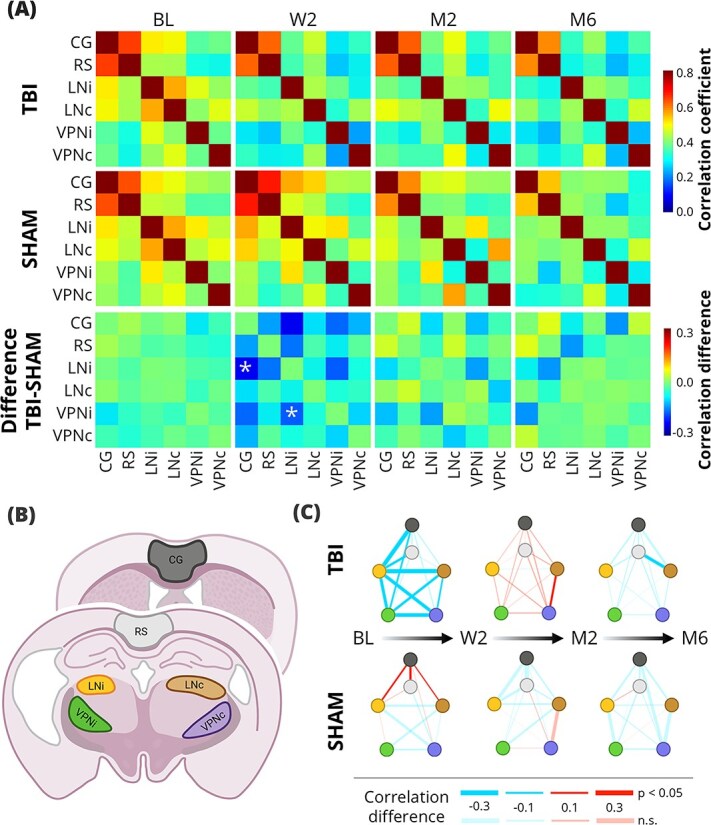
Resting-state fMRI results. (A) the mean FC matrices of the TBI (top row) and SHAM (middle row) animals at all timepoints and the group difference matrices (bottom row); false discovery rate-corrected *t*-test, ^*^q < 0.05. (B) Illustration of the anatomical regions of interest (ROIs). (C) The differences of FCs between timepoints in the TBI (top row) and SHAM (bottom row) group. The color of the line direction of the change in FC; the thickness of the line indicates the size of the difference (see legend). Lines are also coded by transparency to indicate significance: Opaque (non-transparent) lines reflect significant differences (related samples *t*-test, *P* < 0.05), whereas partially transparent lines indicate non-significant comparisons. BL, baseline; CG, cingulate cortical area; LNc, contralateral lateral nuclei; LNi, ipsilateral lateral nuclei; M2, 2 months post-injury; M6, 6 months post-injury; RS, retrosplenial cortical area; VPNc, contralateral ventral posterior nucleus; VPNi, ipsilateral ventral posterior nucleus; W2, 2 weeks post-injury. Partially created in https://www.BioRender.com.

The thalamic areas of interest were selected based on the results from the PET measurements (see Results). The following thalamic areas with increased uptake were selected: the ventral posterior nucleus (VPN), which consists of ventral posterolateral (VPL) and ventral posteromedial (VPM) nuclei; and the lateral nuclei (LN), which consist of laterodorsal (LD) and lateral posterior (LP) nuclei. For the cortical areas, we selected the areas that were outside of the primary injury area: retrosplenial (RS) and cingulate (CG) cortex. The regions of interest (ROIs) were drawn on the reference brain according to an anatomical atlas (Paxinos and Watson 2014). The thalamic areas were drawn ipsilaterally (VPNi, LNi) and contralaterally (VPNc, LNc) to the injury, while the cortical ROIs were drawn bilaterally (RS, CG) as schematically shown in Fig.  2B.

### Functional connectivity

The fMRI data were reconstructed using in-house-created MATLAB codes. In addition to the traditional preprocessing pipeline (slice timing correction and motion correction), we implemented a motion scrubbing and an independent component analysis (ICA)-based approach for motion artifact removal, as previously described (Dvořáková et al. 2021). Scans in which more than 20 out of 30 components were removed in the ICA-based motion-correction step were completely discarded from the FC analysis, resulting in 172 out of 188 scans (8.51% discarded). Framewise displacement metrics (mean and maximum) within the brain mask were estimated from the motion parameters using ANTs (Avants et al. 2009).

Next, we used the anatomical MGE images to coregister the fMRI data. First, the baseline anatomical images were coregistered to the reference brain using affine and nonlinear SyN registration. Next, the consecutive timepoints were registered to their preceding timepoint, i.e. W2 to BL, M2 to W2, and M6 to M2. As the last step, the timepoint co-registrations were applied in a chain so that all images were in register with the reference. Finally, the registration transformations were applied to the functional data.

Functional connectivity analysis was performed on 5-min segments (300 volumes) exhibiting the least motion. The first 5 min were excluded from the analysis to minimize the possible lingering effect of initial anesthesia (Dvořáková et al. 2021). Finally, the motion parameters were regressed from the time series in a voxel-wise fashion. The Pearson correlation of each ROI pair’s mean time series was calculated to assess the FC. To exclude lesion areas or lesion-related artifacts from the ROIs, we first calculated the mean signal across the entire timeseries at each timepoint. After normalizing these mean images for global intensity changes, voxel-wise intensity differences were computed by comparing post-injury to baseline values. Voxels with differences exceeding 2.5 times the overall mean baseline intensity (averaged across all animals) were excluded. ROIs with more than 30% excluded voxels were omitted from the fMRI analysis.

### Structural connectivity

DTI data were processed using a computational framework for bundle-specific tractography established for the EpiBioS4Rx project (Manninen et al. 2025; Sharma et al. 2025). We performed tractography to confirm the structural connectivity between CG and LN and to establish a mid-tract ROI for subsequent analysis. We created two prototypical bundles connecting the CG and LN areas, in the ipsilateral (CG-LNi) and the contralateral (CG-LNc) sides. Next, we performed the streamline estimations of both tracts in each scan. Tractography was unsuccessful in 9.78% of the tracts due to technical issues. The prototypical curve of each prototypical bundle was divided into 8 segments, and for the ROI analysis, the 4 segments in the caudate putamen were used. The prototypical curves were projected to the individual scans, and the voxels that contained the streamlines were included in the ROI. As this part mainly included the dorsal medial striatum, it is noted here as DMSi for the ipsilateral area and DMSc for the contralateral area. The DTI-related metrics: fractional anisotropy (FA), mean diffusivity (MD), radial diffusivity (RD), and axial diffusivity (AD), were computed, and the mean values of the 4 segments were calculated. Additionally, the ROI set that was used in the fMRI analysis (LNi, LNc, VLNi, VLNc, RS, CG) was projected to the DTI maps, and the mean value for each DTI-related metric in each ROI was computed.

### Positron emission tomography

The PET data were reconstructed using the OSEM-2D algorithm, and the frames between 15 and 60 min post-injection were summed for the activity quantification. The PET data were manually coregistered to the CT images and anatomical MGE images acquired on W2 using Carimas 2.10 (Rainio et al. 2023). Based on the time course of the tracer activity, scans with a faulty tail vein injection were excluded (7 TBI, 1 SHAM), which resulted in a total of 26 successful scans (18 TBI, 8 SHAM). The values of radioactivity concentrations were normalized to the uptake in the part of the body visible in the field of view (rostro-thoracic region). The activity maps and ROI values are presented as a percentage of the total rostro-thoracic uptake per ml (%/ml).

### Histological analyses

High-resolution photomicrographs of Nissl and myelin-stained sections were acquired using a VS200 research slide scanner (Olympus, Evident Corporation, Shinjuku, Tokyo, Japan). We first identified 12 consecutive sections that cover the analyzed thalamic ROIs in in vivo imaging analyses, and from those, we selected 3 representative sections spaced to cover the thalamic ROI. The same thalamic areas (LNi, LNc, VPNi, VPNc) and, additionally, the unilateral RS areas (RSi, RSc) were manually drawn on the selected sections. Animals in which severe cortical damage occurred as an unintended consequence of the EEG electrode implantation were excluded from the ROI study. The total number of animals with histological analyses was 20 (14 TBI, 6 SHAM).

The Nissl-stained sections were analyzed with Qupath, where the watershed cell detection algorithm was used to segment the cells. The cell densities of neuronal (CD_ne_) and glial (CD_gl_) cells were determined based on the size of nuclei. To establish classification thresholds, we have manually selected 731 glial cells and 966 neuronal cells based on their morphological features across all ROIs and levels in both TBI and SHAM animals. The distribution of the nuclear areas is presented in Supplementary Fig. 1. Thresholds for classification were defined as the mean value ±2 standard deviations for each manually selected cell population. Neuronal cell nuclei ranged from 52 to 215 μm^2^, and the glial nuclei size from 8 to 34 μm^2^. All the detected cells were then classified according to these thresholds. The cell density (CD) was estimated in each selected section for neuronal and glial cells as: $CD=\frac{CC}{ROI_{area}},$ where $CC$ is either the neuronal or glial cell count in the ROI and ${ROI}_{area},$ is the area of the ROI. The average values across the three sections were considered in further analysis.

The optical density (OD) of myelin-stained sections was quantified using Qupath in the consecutive sections, as in the cell detection analysis. The OD was corrected for the possible difference in staining. The corrected OD was estimated as: $OD=\frac{I_{ROI}-{I}_{BG}}{I_{WM}},$ where ${I}_{ROI}$ is the raw optical density of each ROI, ${I}_{BG}$ is the reference of the background intensity taken in the dentate gyrus in an area with no cells or myelin, and ${I}_{WM}$ is the reference values of high myelin content taken in the optical tracts. All the reference values (${I}_{WM}$ and ${I}_{BG}$) were taken from the same section as their respective ${I}_{ROI}$. The OD of each ROI was estimated in each section, and the mean value across the three sections was calculated.

### Statistical analysis

For all the statistical analysis of the FC, the Fisher z-transfor-mation was applied to the correlation values. To test group differences between TBI and SHAM of the FC and ROI measures (FA, RD, AD, MD, CD_ne_, CD_gl_, OD, U_FEPPA_), a two-sample *t*-test was used. To assess the differences of the FC between timepoints in each group and to test the ipsilateral-contralateral differences of the thalamic nuclei (and the RS in histological results), a paired *t*-test was used. All *P*-values that were false discovery rate (FDR)-corrected are noted as q. Values of *P* less than 0.05 and of q less than 0.05 were considered statistically significant. The framewise displacement values are presented as mean ± standard deviation.

Next, we explored the relation of the sub-acute uptake of the neuroinflammation marker and the lateral alterations of FC, DTI, and histological parameters. To reduce intersubject variability, we calculated differences between the ipsilateral and contralateral thalamic nuclei ROI values, noted as Δ (eg ΔOD[LN] = OD[LNi]—OD[LNc]). For the FC, we calculated the difference as ΔFC_CG_(LN) = FC_CG-LNi_—FC_CG-LNc_, and similarly also for ΔFC_RS_(LN), ΔFC_CG_(VPN), and ΔFC_RS_(VPN). Spearman correlation coefficients were computed between the ΔU_FEPPA_ and the lateral difference in the diffusion metrics (ΔFA, ΔRD, ΔAD, ΔMD), histological measures (ΔCD_ne_, ΔCD_gl_), and the fMRI correlation values (ΔFC_CG_, ΔFC_RS_) in each thalamic nucleus. Both TBI and SHAM animals were included in this analysis. The correlation values with *P*-values lower than 0.05 were considered significant.

## Results

The primary lesion was located in the caudal lateral cortical areas, including the auditory, primary visual, temporal association, primary, and secondary somatosensory cortical areas (Supplementary Fig. 2). Injury and lesion progression as observed in anatomical images are shown in Fig.  1B. At W2, ventricle size increased ipsilaterally to the injury. By M2, the lesion markedly progressed, resulting in cortical thinning in the area of the primary lesion, accompanied by increased ventricle sizes in both hemispheres and atrophy of the ipsilateral hippocampus and thalamus. The lesion still progressed until M6, although the changes between M2 and M6 were less pronounced. The lesion showed some heterogeneity in size and location across animals (Supplementary Fig. 3).

### Functional connectivity

The mean framewise-displacement (FD) derived from the motion correction parameters across all measurements was 0.028 ± 0.022 mm, and the mean value of the maximum FD within the 300-volume window was 0.168 ± 0.105 mm. There were no differences in the mean FD between TBI and SHAM animals at any timepoints (Supplementary Fig. 4). Notably, FD did not increase from the baseline values, suggesting that the two-day habituation protocol before the subsequent timepoints was effective.

The FC analysis results are shown in Fig.  2. At baseline, there were no significant differences between the two groups. Following the injury, the TBI group showed a trend towards decreased overall FC compared to the SHAM group, with connectivity in CG-LNi and LNi-VPNi showing a significant difference (q < 0.05). Although the hypoconnectivity of the ipsilateral thalamic areas appeared to also persist at M2, the group-level differences were no longer statistically significant. By the M6, the thalamo-thalamic FC differences had resolved, with group-level differences in correlation values being < 0.05. However, mild non-significant hypoconnectivity in the ipsilateral corticothalamic FC appeared to remain.

We then assessed differences in FC between timepoints in each group (Fig.  2C). In the TBI group, the thalamo-thalamic FC decreased at W2 (*P* < 0.05). While both ipsilateral and contralateral corticothalamic connectivity also decreased at W2 compared to the BL, only the ipsilateral decrease was significant (*P* < 0.05). From W2 to M2, there was a modest increase in overall FC, with only LNc-VPNc reaching statistical significance (*P* < 0.05). Between the M2 and the M6, there was a slight global decrease in FC, but only the RS-LNc decreased significantly (*P* < 0.05). In the sham-operated group, there was a significant increase in RS-CG, RS-LNc, and RS-LNi connectivity between the BL and W2 (*P* < 0.05), which could be a result of the craniotomy, with no other significant changes across timepoints.

Additionally, we tested for ipsilateral-contralateral differences of the corticothalamic FC across the timepoints (Supplementary Table 1). In the TBI group, no significant difference was found at BL (q > 0.05). Following the injury, the ipsilateral connections were generally weaker compared to the contralateral ones. The lateral differences were significant for CG-LN connections at all post-injury timepoints, and for CG-VPN at M2 and M6 (q < 0.05). Significant differences in VPN-CG were also observed at M2 and M6 (q < 0.05), while no differences were detected for VPN-RS (q > 0.05). No statistically significant lateral differences were found in the SHAM group at any timepoint. Overall, we observed alterations of FC as a result of the injury, which were mostly resolved by M6. Nevertheless, some lateral differences of the corticothalamic FCs still remained.

### Positron emission tomography

**Fig. 3:**
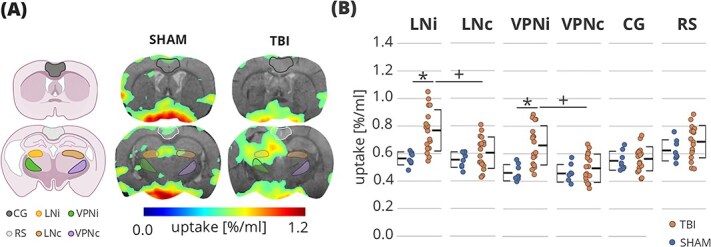
PET data analysis. (A) The uptake of [^18^F]-FEPPA in representative TBI (right) and SHAM (middle) animals, and the anatomical ROIs (left). (B) The ROI analysis of [^18^F]-FEPPA uptake. Statistical significance: + indicates *q*  < 0.05 between ipsilateral and contralateral thalamic nuclei in a false discovery rate-corrected two-sample *t*-test; ^*^ indicates *q* < 0.05 between the SHAM and TBI animals in a false discovery rade-corrected related *t*-test. Error bars show the standard deviation. CG, cingulate cortical area; LNc, contralateral lateral nuclei; LNi, ipsilateral lateral nuclei; RS, retrosplenial cortical area; VPNc, contralateral ventral posterior nucleus; VPNi, ipsilateral ventral posterior nucleus. Partially created in https://www.BioRender.com.

Increased binding of the TSPO tracer [^18^F]-FEPPA in the TBI animals was observed in the perilesional cortical areas and ipsilateral subcortical regions such as the thalamus and hippocampus (Fig.  3A). Elevated [^18^F]-FEPPA uptake was also noted in the cerebellum and around large blood vessels across all the animals, consistent with the known physiological distribution of TSPO (Supplementary Fig. 5). The ROI analysis (Fig.  3B) demonstrated significantly higher tracer binding in the LNi and VPNi compared to their contralateral counterparts (q < 0.05, Supplementary Table 2). Additionally, the uptake in the ipsilateral thalamic nuclei in the TBI group was higher than in the SHAM group (q < 0.05), with no significant differences in the cortical areas (q > 0.05). Given that the expression of TSPO is located in the outer mitochondrial membrane of reactive glia cells, these findings indicate thalamic neuroinflammation in the ipsilateral thalamic nuclei at W2 post-injury.

### Diffusion tensor imaging

**Fig. 4:**
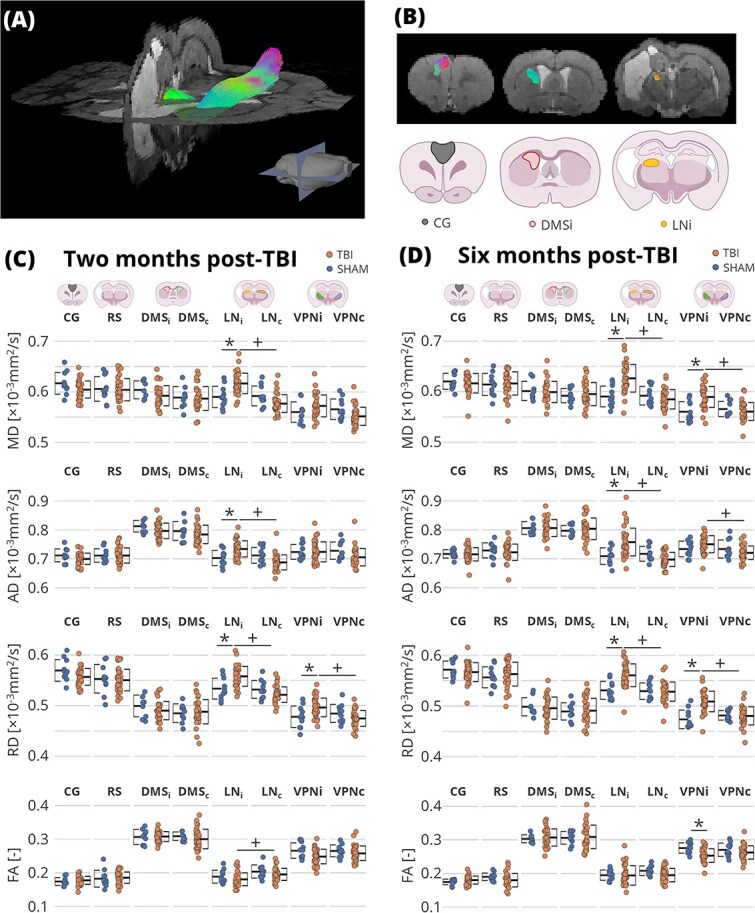
DTI analysis results and CG-LN tractography. (A) 3D representative example of streamlines of the CG-LN pathway in the ipsilateral side of a TBI animal at six months post-injury (B) 2D views of the streamlines in the ipsilateral side at CG (left), DMS (center), and LN (right) level. (C, D) The ROI analysis of the diffusion metrics at two months (C) and six months (D) post-injury. Error bars show the standard deviation. Statistical significance: + indicates *q* < 0.05 between ipsilateral and contralateral thalamic nuclei in a false discovery rate-corrected two-sample *t*-test; ^*^ indicates *q*  < 0.05 between the SHAM and TBI animals in a false discovery rate-corrected related *t*-test. AD, axial diffusivity; CG, cingulate cortical area; FA, fractional anisotropy; LNc, contralateral lateral nuclei; LNi, ipsilateral lateral nuclei; MD, mean diffusivity; RD, radial diffusivity; RS, retrosplenial cortical area; VPNc, contralateral ventral posterior nucleus; VPNi, ipsilateral ventral posterior nucleus. Partially created in https://www.BioRender.com.

An example of the estimated tract connecting the LN and CG, projecting through the dorsal medial striatum (DMS), is shown in Fig.  4A, and the schematic example of the DMS ROI derived from the tractography is presented in Fig.  4B. First, we analyzed the three nodes of the CG-LN pathways: cortical (CG), striatal (DMS), and thalamic (VN) nodes (Fig.  4C and D). The ROI analysis revealed no alterations of DTI-metrics in the CG and DMS (q > 0.05), indicating that these two nodes did not sustain chronic TBI-related structural damage. However, diffusivity measures (MD, AD, and RD) were increased in LNi at both M2 and M6 compared to the SHAM (q < 0.05). Next, we analyzed RS and VPN ROIs (Fig.  4C and D). We found no statistical differences in RS and VPNc (q > 0.05). While no statistical differences were observed between TBI and SHAM in VPNi at M2, we did observe increased MD and RD and reduced FA at M6 (q < 0.05).

In the analysis of ipsilateral-contralateral differences (Supplementary Table 3), TBI animals exhibited higher diffusivity measures (MD, AD, and RD) in both ipsilateral thalamic areas compared to their contralateral counterparts at both M2 and M6 (q < 0.05), except for AD in VPN at M2, where no difference was observed (q > 0.05). Additionally, FA was lower in LNi relative to LNc at 2 M (q < 0.05). No lateral differences were found in the DMS, CG, and RS in the TBI group, nor in any ROI in the SHAM group (q > 0.05). Collectively, these findings suggest persistent structural alterations in the ipsilateral thalamus following TBI, while other segments of the CG-LN pathway remained unaffected.

### Histology

**Fig. 5:**
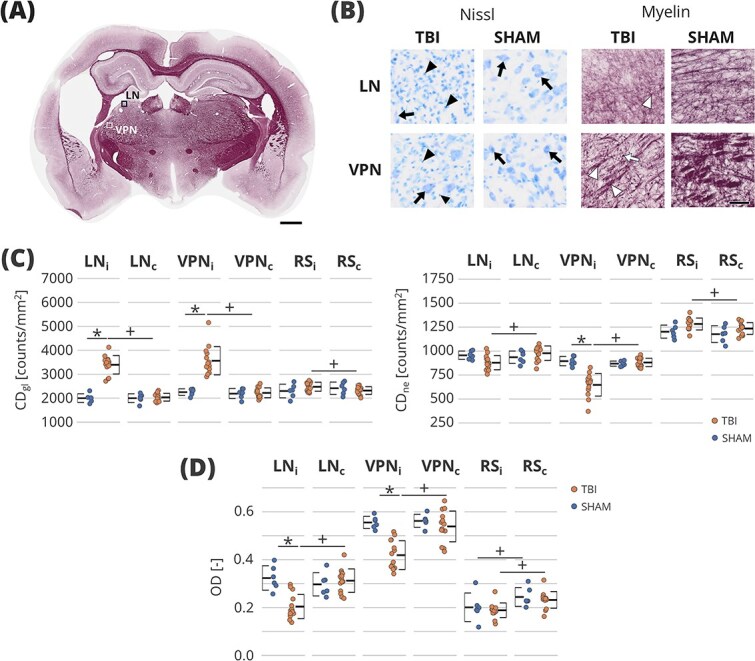
Results of histological analysis. (A) Representative whole-brain myelin-stained section. The squares denote the location of photomicrographs with higher magnification of both myelin-stained and Nissl-stained sections of both TBI and SHAM-operated animals in (B). Black arrowheads point to gliosis, black arrows point to neuronal cells, white arrowheads indicate a decrease in myelin density, and the white arrow shows axonal damage. (C) ROI analysis of Nissl-stained sections. (D) ROI analysis of myelin-stained section. Statistical significance: + indicates *q* < 0.05 between ipsilateral and contralateral thalamic nuclei in a false discovery rate-corrected two-sample *t*-test; ^*^ indicates *q* < 0.05 between the SHAM and TBI animals in a false discovery rate-corrected related *t*-test. Error bars show the standard deviation. Scale bars: 1 mm (A), 50 μm (B). CD_gl_, cell density of glial cells; CD_ne_, cell density of neuronal cells; CG, cingulate cortical area; d, cell diameter; LN, lateral nuclei; LNc, contralateral lateral nuclei; LNi, ipsilateral lateral nuclei; OD, optical density; RSi, ipsilateral retrosplenial cortical area; RSc, contralateral retrosplenial cortical area; VPN, ventral posterior nucleus; VPNc, contralateral ventral posterior nucleus; VPNi, ipsilateral ventral posterior nucleus.

Representative histological photomicrographs and ROI-based analyses are shown in Fig.  5. Nissl-stained sections showed increased cellularity and gliosis in the ipsilateral thalamus after TBI. ROI analysis (Fig.  5C, Supplementary Table 4) demonstrated an increase of CD_gl_ in the ipsilateral thalamic nuclei in the TBI group compared to the SHAM group (q < 0.05). Moreover, the ipsilateral thalamic nuclei had statistically significantly higher CD_gl_ than their contralateral counterparts. Interestingly, although we have observed a decreased CD_ne_ in both ipsilateral thalamic nuclei compared to the ipsilateral side in TBI animals, only VPNi showed a significant decrease in CD_ne_ in the TBI group compared to the SHAM (q < 0.05). Additionally, we have observed a significant difference in the TBI group between the ipsilateral and contralateral RS ROI in both CD_gl_ and CD_ne_ (q < 0.05).

In the myelin staining, both ipsilateral thalamic nuclei (VNi, VPNi) were atrophied and showed disrupted axonal organization, axonal damage, and myelin loss after TBI (Fig.  5B). ROI-based analysis (Fig.  5D, Supplementary Table 4) showed lower OD of the myelin staining in the TBI group compared to the SHAM group. Moreover, the ipsilateral thalamic nuclei showed lower OD compared to their contralateral counterparts in the TBI group. Interestingly, lateral differences were also found in the cortical ROI of both TBI and SHAM animals.

**Fig. 6:**
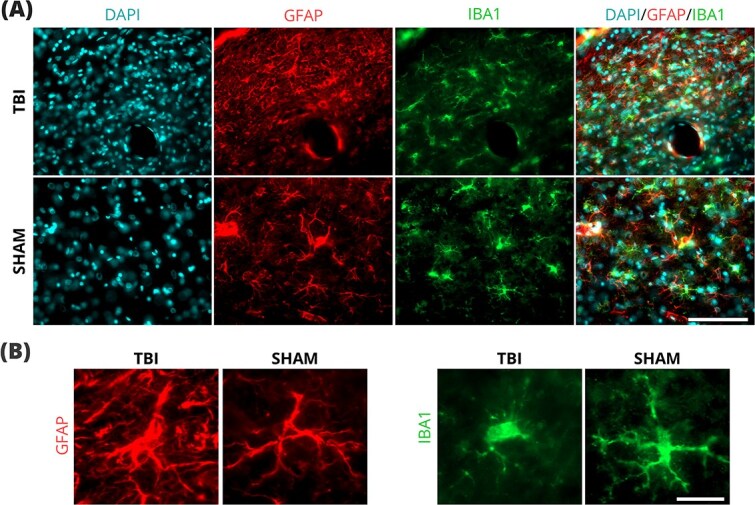
(A) Representative section of LN labeled with DfAPI, GFAP, and IBA1. TBI animals showed higher cellularity (DAPI) and immunoreactivity to GFAP and IBA1. (B) Representative examples of astrocytes (GFAP) and microglia (IBA1) in SHAM and TBI animals. Scale bars: 100 μm (A), 20 μm (B). GFAP, glial fibrillary acidic protein; IBA1, ionized calcium-binding adaptor molecule 1; TBI, traumatic brain injury.

Immunohistochemical labeling supported the Nissl staining findings in thalamic areas (Fig.  6, Supplementary Fig. 6). TBI samples exhibited increased cell density and enhanced immunoreactivity for both IBA1 and GFAP in LNi and VPNi. Moreover, the TBI samples showed microglia with morphology typical of activated cells. Similarly, reactive astrocytes were found in the TBI samples. These results point to chronic thalamic neuroinflammation at 8 months post-TBI.

### Association of the [^18^F]-FEPPA uptake with lateral asymmetry

**Fig. 7:**
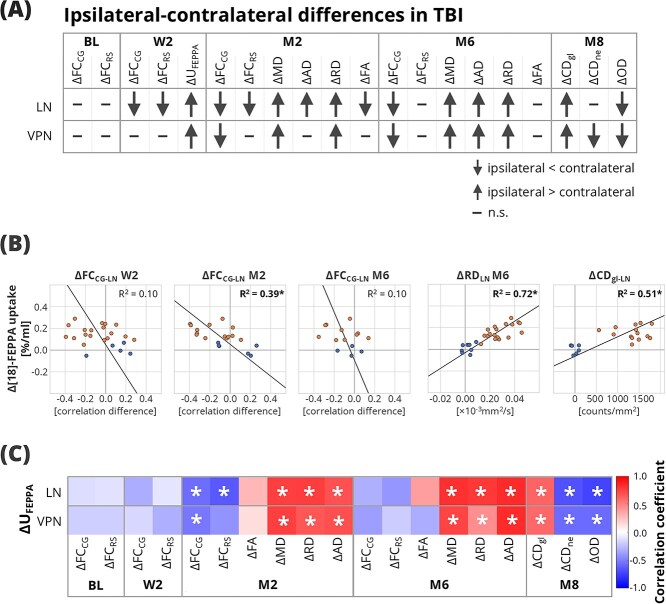
(A) Overview of the observed ipsilateral-contralateral differences across all the modalities and timepoints in TBI animals in LN and VPN. (B) Scatter plots of the ΔU_FEPPA_ in LN and corresponding differences of in other imaging and histological metrics and their respective linear regression line. Asterisk (^*^) denotes statistically significant linear regressions (q < 0.05). (C) Spearman correlation coefficients between the ΔU_FEPPA_ in LN and VPN and corresponding differences of other imaging and histological metrics, asterisk (^*^) denotes statistically significant correlations (*P* < 0.05). AD, axial diffusivity; BL, baseline; CD_gl_, cell density of glial cells; CD_ne_, cell density of neuronal cells; FA, fractional anisotropy; FC_CG_, thalamic functional connectivity to cingulate cortical area; FC_CG-LN_, functional connectivity of the lateral nuclei to cingulate cortical area; FC_RS_, thalamic functional connectivity to retrosplenial cortical area; LN, lateral nuclei; MD, mean diffusivity; n.s.; non-significant; M2, 2 months post-injury; M6, six months post-injury; M8, eight months post-injury; OD, optical density; RD, radial diffusivity; U_FEPPA_, uptake of the [^18^F]-FEPPA; VPN, ventral posterior nucleus, W2, 2 weeks post-injury.

Across all modalities, we consistently observed significant lateral asymmetry in both thalamic nuclei in diffusivity parameters, neuroinflammatory marker uptake, corticothalamic connectivity, and histological measures (see overview in Fig.  7A). To investigate whether these changes relate to neuroinflammation, we performed Spearman correlation analysis between the ΔU_FEPPA_ in LN and VPN and corresponding differences in other imaging and histological metrics (overview in Fig.  7C, representative examples of scatterplots in Fig.  7B and all scatterplots shown in Supplementary Figs. 7 and 8). The results revealed strong correlations between the ΔU_FEPPA_ and the diffusivity differences (ΔAD, ΔMD, ΔRD) at both M2 and M6. Additionally, ΔU_FEPPA_ also correlated well with the histologic metrics differences (ΔCD_ne_, ΔCD_gl_, ΔOD) in both thalamic nuclei. Among the FC metrics, only those at the M2 timepoint showed a statistically significant correlation to the ΔU_FEPPA_, specifically for the CG-VPN, CG-LN, and RS-LN connections. Taken together, these findings suggest that subacute neuroinflammatory changes are closely associated with long-lasting structural, microstructural, and functional lateral thalamic alterations.

## Discussion

Here, we presented the first longitudinal study following the interrelationship of neuroinflammation and changes in the corticothalamic connectivity after TBI in rats. We observed initial hypoconnectivity in the ipsilateral corticothalamic connectivity after injury, which mainly resolved by 6 months post-TBI. DTI further revealed altered diffusivity within the ipsilateral thalamus at chronic stages. Additionally, we detected secondary thalamic inflammation, which correlated with the lateralized changes observed in this study, and this inflammation persisted up to 8 months post-injury, as confirmed by histological analysis.

Our findings of secondary thalamic inflammation are consistent with the hypothesis that neuroinflammatory dissemination follows the cortico-thalamo-cortical circuits (Necula et al. 2022). Specifically, the thalamic nuclei topographically connected to the initial cortical lesion exhibit secondary inflammation (Necula et al. 2022). In our model, the primary injury extended rostro-caudally over multiple cortical areas. The caudal lesion areas included primary and higher-order visual areas, which connect to the LN, one of the visual thalamic relay areas (Vertes et al. 2015). Rostral lesion areas included sensorimotor cortical areas, which are densely interconnected with VPN (Vertes et al. 2015). Accordingly, we observed inflammation in both LN and VPN. This finding strengthens previous observations in mouse models (Langen et al. 2007; Necula et al. 2022).

Although our study did not include behavioral assessments, the pattern of thalamic inflammation is consistent with several commonly reported behavioral outcomes in both animal models and TBI patients. Specifically, the observed chronic inflammation in VPN, which is involved in somatosensory function and pain perception (Guilbaud et al. 1990; Berkley et al. 1993; Purves and Williams 2001; Lipshetz et al. 2018), corresponds to reports of chronic peripheral pain sensitivity and impaired sensorimotor function after LFPI in rats (Stelfa et al. 2022). Sensorimotor impairments have also been observed in rats and mice after controlled cortical impact (CCI) (Onyszchuk et al. 2007; Schönfeld et al. 2017), in certain blast TBI studies (Aravind et al. 2020), and in TBI patients (Galea et al. 2018, 2022). In addition, we observed chronic inflammation in LN, which has been implicated in visual attention processes in rats (Kamishina et al. 2009). Consistently, sustained attention deficits have been reported in rats following CCI (Kutash et al. 2023). Similarly, the homologous human thalamic structure, the pulvinar, is associated with attention (Arcaro et al. 2018), which is often impaired in patients post-TBI (Arcaro et al. 2018). Together, these observations suggest that chronic thalamic inflammation may be a possible contributor to the functional disturbances observed following TBI.

At the subacute timepoint (W2), TBI animals showed initial hypoconnectivity across all connections compared to SHAM animals, but these differences were not present at later stages. This pattern aligns with findings from Vinh To et al. (2022) who reported global hypoconnectivity one day post-injury but found no injury-related differences in FC seven days post-injury in a mouse model of CCI (Vinh To et al. 2022). Similarly, Mohamed et al. also reported the lowest connectivity at the subacute timepoint (seven days post-injury) in a rat CCI model (Mohamed et al. 2021). Interestingly, in their study, the contralateral-contralateral connectivity then increased at 60 days post-injury and was slightly reduced by six months. We observed a similar trajectory: recovery of the contralateral thalamic FCs between two weeks and two months in TBI animals, which then slightly decreased by six months to the baseline levels. Together, these results highlight a potentially consistent trajectory of FC alterations following TBI, regardless of species or injury model.

We observed increased diffusivity parameters (MD, AD, and RD) in both thalamic nuclei, consistent with our previous findings in the same model at six months post-TBI (Laitinen et al. 2015). However, in our previous subacute timepoint study (at 7- and 21-days post-injury), diffusivity parameters were decreased ipsilaterally (Manninen et al. 2021). A similar time-dependent biphasic response of the diffusive parameters to the injury has been reported in the CCI model (Soni et al. 2019; Mohamed et al. 2021). Early decreases of diffusivity are thought to reflect the cytotoxic edema, characterized by cellular swelling and reduced extracellular space for water diffusion (Soni et al. 2019; Amlerova et al. 2024). In contrast, vasogenic edema corresponds to extracellular fluid accumulation, which is reflected by high diffusivity (Soni et al. 2019; Kulkarni et al. 2020). Apart from the edema, neuroinflammation has also been proven to affect the diffusivity measures, as gliosis affects the diffusion (Budde et al. 2011). Additionally, the features of the secondary injury, such as demyelination, axonal degeneration, and axonal swelling, also affect the diffusivity measures (Song et al. 2005; Aung et al. 2013; Hayes et al. 2016; Holikova et al. 2021). In our study, the lateral differences of diffusivity correlated well with the subacute inflammation. However, as we have observed both signs of inflammation and axonal damage in histology, the exact source of the diffusivity-related alterations remains unclear and is most likely a combined effect of the TBI-related pathophysiology.

Neuroinflammation is characterized by activation of glial cells: microglia and astrocytes, both of which play an important role in the synaptic activity and homeostasis (Müller et al. 2025). Additionally, a recent study utilizing the functional ultrasound in lipopolysaccharide-induced neuroinflammation showed inflammation-related high oscillations of spontaneous hemodynamic changes and high fractional amplitude of low-frequency fluctuation (fALFF) values in the affected areas (Pereira et al. 2025), though we did not observe such increase in the thalamic regions (fALFF data not shown). In our study, however, the lateral changes of the subacute neuroinflammation correlated with the lateral alterations of the FC only at two months post-injury. At six months, this correlation was weaker, potentially due to neuroplasticity and compensatory mechanisms, which have been observed both in humans and rodents (Hylin et al. 2017; Kashou et al. 2024). Interestingly, even at the subacute timepoint, despite the fMRI and PET measurements being only five days apart, the correlation between neuroinflammation and FC was weak, although this timepoint exhibited the most pronounced FC changes. The consequences of the TBI are highly complex, with region-dependent mechanisms, such as edema, cell apoptosis, blood–brain barrier (BBB) dysfunction, and focal microbleed unfolding over distinct timelines (Glushakov et al. 2018; Soni et al. 2019; van Vliet et al. 2020; van der Eerden et al. 2022). It is therefore possible that the observed changes in FC may be influenced by some of these overlapping processes, contributing to the weak association with neuroinflammation at W2. The exact underlying processes and their timing in our study, however, remain unclear, as our study specifically focuses on neuroinflammation. It is important to consider, however, that the spatial resolution of both fMRI and PET is relatively low, and the selected regions of interest consist of multiple thalamic nuclei, potentially obscuring finer details of injury progression.

The primary focus of this study was the corticothalamic functional connectivity, which is known to be sensitive to the effects of anesthesia. Prior preclinical and clinical fMRI studies have shown that corticothalamic connectivity is suppressed by various routinely used anesthetics, including isoflurane (Liang et al. 2013; Bukhari et al. 2017; Paasonen et al. 2018), medetomidine (Bukhari et al. 2017; Paasonen et al. 2018), dexmedetomidine (Kantonen et al. 2023), and propofol (Paasonen et al. 2018; Kantonen et al. 2023). To mitigate the confounding effect of anesthesia, we have previously introduced a light sedation protocol in habituated, body-restrained rats (Dvořáková et al. 2021). Although some of the thalamic connectivity to lateral cortical areas differed from the awake state, the thalamic connectivity to cingulate and retrosplenial areas remained virtually unchanged (Dvořáková et al. 2021). This light sedation approach preserves FC while reducing animal movement compared to the awake studies.

### Limitations of the study

An unexpected finding was the reduced optical density of myelin-stained sections and the altered cell density in ipsilateral retrosplenial cortical areas, which are outside the primary lesion area. This may be due to widespread subtle changes in this model or coming from experimental imperfections. To ensure orientation during histological processing, a unilateral cortical branding mark was applied to the ipsilateral hemisphere. This marking caused localized alterations in tissue morphology, which resulted in staining artifacts in the affected cortical area in both groups. Despite efforts to exclude the marked region during manual ROI definition, it is possible that some affected tissue was inadvertently included in the ROI, potentially contributing to the observed lateral asymmetry in measurements. While there is some evidence of longitudinal structural changes in the cortical regions distant from the initial lesion at chronic timepoints (Soni et al. 2019; Mohamed et al. 2021), the fact that the histological findings in the cortical areas were present in both TBI and SHAM groups suggests that a systematic error is a more likely explanation.

In this study, we quantified two markers of neuroinflammation: a TSPO tracer at 2 weeks post-TBI and Nissl-derived glial cell density at 8 months. Both neuroinflammatory markers lack specificity in distinguishing between neurotoxic and neuroprotective inflammatory states. TSPO is a non-specific neuroinflammatory marker expressed by activated astrocytes, microglia, and other inflammatory cells, both in pro- and anti-inflammatory states. Although it has been traditionally described as a microglia-specific tracer, increasing evidence suggests that TSPO expression extends to multiple cell types involved in neuroinflammation, indicating that it represents a general marker of inflammatory activity (Lavisse et al. 2012; Wimberley et al. 2018; Pannell et al. 2020; Delage et al. 2021). Similarly, the Nissl-derived glial cell density is a general neuroinflammatory marker, as Nissl staining binds to the nucleic acids regardless of cell type. The measured cell density therefore includes microglia, astrocytes, and oligodendrocytes, as well possible non-glial cells (eg endothelial cells and leukocytes). Given that glial cells constitute the majority, and our immunohistochemical qualitative analyses demonstrated increased astrocytic and microglial labeling, we interpret the observed increase in Nissl-derived cell density as reflecting glial proliferation. While we confirmed activated microglia and astrocytes through immunochemistry labeling, differentiation between pro- and anti-inflammatory phenotypes was not feasible. Although previous preclinical studies have demonstrated that longitudinal inflammatory responses are predominantly neurotoxic, future studies would benefit from incorporating markers that can differentiate glial phenotypes.

Despite using a light sedation protocol, animals were exposed to isoflurane during multiple imaging sessions and briefly during habituation. Given its documented anti-inflammatory effects, isoflurane may have affected our results (Wu et al. 2010). However, as it has also been shown that isoflurane can also promote neuroinflammatory processes, its overall effect in this study is uncertain (Lee et al. 2015; Cruz et al. 2017). Additionally, the TBI causes GABA receptor channelopathy in thalamic areas (Drexel et al. 2015). Hence, the effect of isoflurane, a GABA agonist, potentially creates differential impacts between TBI and SHAM animals. However, the low isoflurane concentration (0.5%) used during the fMRI measurement likely minimized major confounding effects.

Another consideration is the possible presence of iron deposits and calcifications in the thalamic areas after TBI, which can create local magnetic field inhomogeneities (Lehto et al. 2012; Laitinen et al. 2015). As the echo-planar read-out used here for both fMRI and DTI is sensitive to such variations, these could influence our diffusion and functional imaging results (Laitinen et al. 2015).

Lastly, the main focus of our study is the corticothalamic connectivity and therefore any alterations to the large-scale brain networks were not explored and warrant further investigation.

## Conclusion

Overall, our results underscore the importance of thalamic neuroinflammation in shaping both the functional and structural corticothalamic connectivity after TBI. The dynamic evolution of connectivity and diffusivity parameters highlights the importance of longitudinal approaches in understanding secondary injury mechanisms.

## Supplementary Material

Supplementary_Figures_bhaf337

Supplementary_Methods

Supplementary_Tables_(1)_bhaf337

## Data Availability

The data and software are available at: https://doi.org/10.23729/fd-2bdd220c-fcd5-352e-a37f-03aac2f66ecf.
