# Supplementary material for: Secondary thalamic neuroinflammation associates with disturbed corticothalamic connectivity in a model of severe traumatic brain injury in male rats—a longitudinal study
Source: Cereb Cortex. 2026 Jan 9;36(1):bhaf337. doi: 10.1093/cercor/bhaf337 (PMC12784941; doi:10.1093/cercor/bhaf337)
Supplement: Supplementary_Figures_bhaf337 [file supplementary_figures_bhaf337.docx]

**Supplementary Figures**

Secondary thalamic neuroinflammation associates with disturbed corticothalamic connectivity in a model of severe traumatic brain injury in male rats – a longitudinal study

Lenka Dvořáková^1^, Raimo A. Salo^1^, Petteri Stenroos^1^, Kimmo Jokivarsi^1^, Jenni Kyyriäinen^1^, Ekaterina Paasonen^1,2^, Eppu Manninen^1^, Mikko Kettunen^1^, Pekka Poutiainen^3^, Alejandra Sierra^1^, Jaakko Paasonen^1^ and Olli Gröhn^1^*

*^1^A. I. Virtanen Institute for Molecular Sciences,* *University of Eastern Finland, Kuopio, Finland*

*^2^Neurocenter*, *Kuopio University Hospital, Kuopio, Finland*

*^3^Diagnostic Imaging Center, Kuopio University Hospital, Kuopio, Finland*

*Corresponding Author:

Professor Olli Gröhn, Ph.D.

e-mail: [olli.grohn@uef.fi](mailto:olli.grohn@uef.fi)

telephone: +358 50 3590963

mailing address:
A.I.V. Institute for Molecular Sciences, University of Eastern Finland
P.O. Box 1627
Neulaniementie 2,
FI-70211, Kuopio
Finland


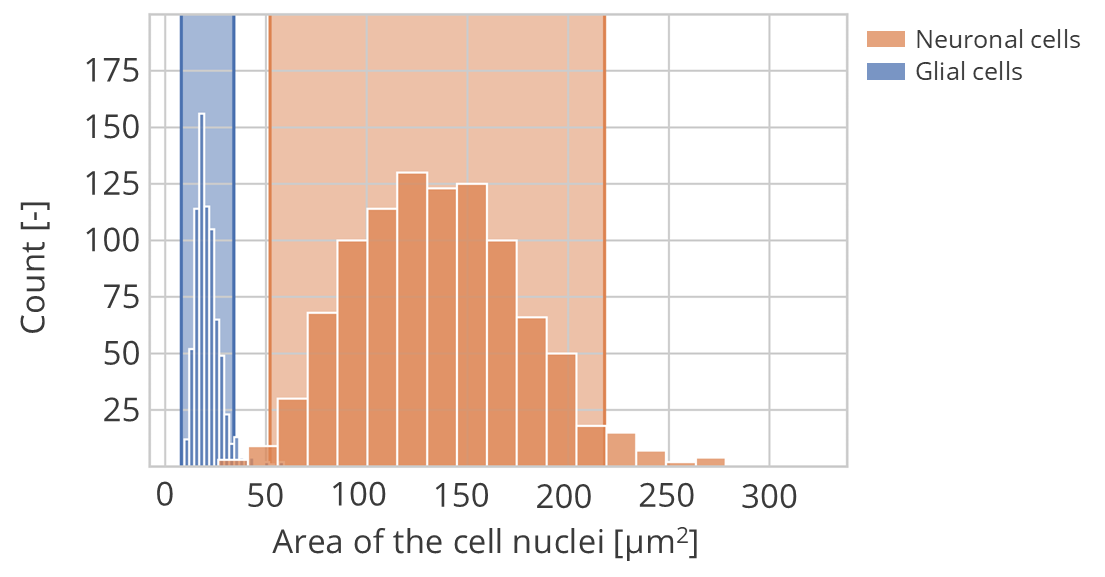


**Supplementary Figure 1**: The distribution of the cell nuclei sizes in the manually selected groups of glial cells (blue) and neuronal cells (orange). The thresholds that were used to categorize the rest of the detected cells are shown as colour bands.

*
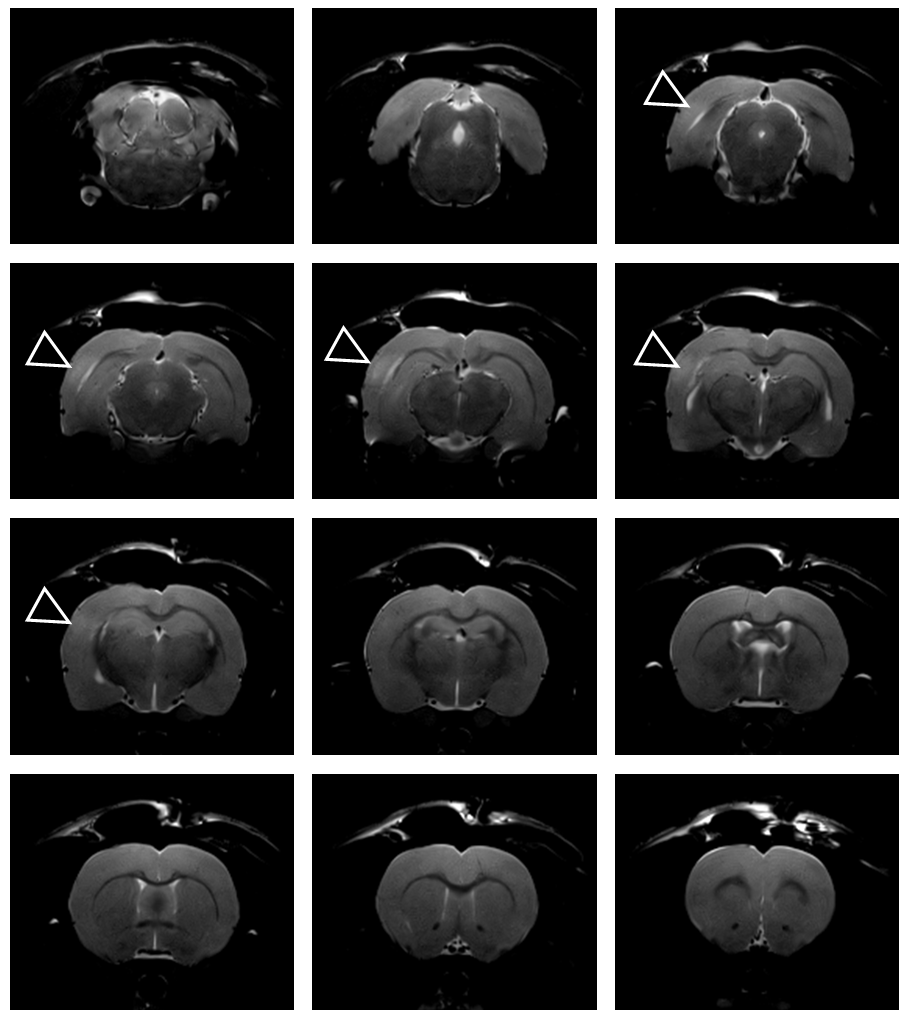
*

**Supplementary Figure 2**: Representative example of the initial lesion in one animal, T2-weighted anatomical images taken at two days post-injury. The arrowhead points to the cortical lesion that shows as a hyperintense signal. The images were denoised using the ANTs spatially adaptive filter tool.


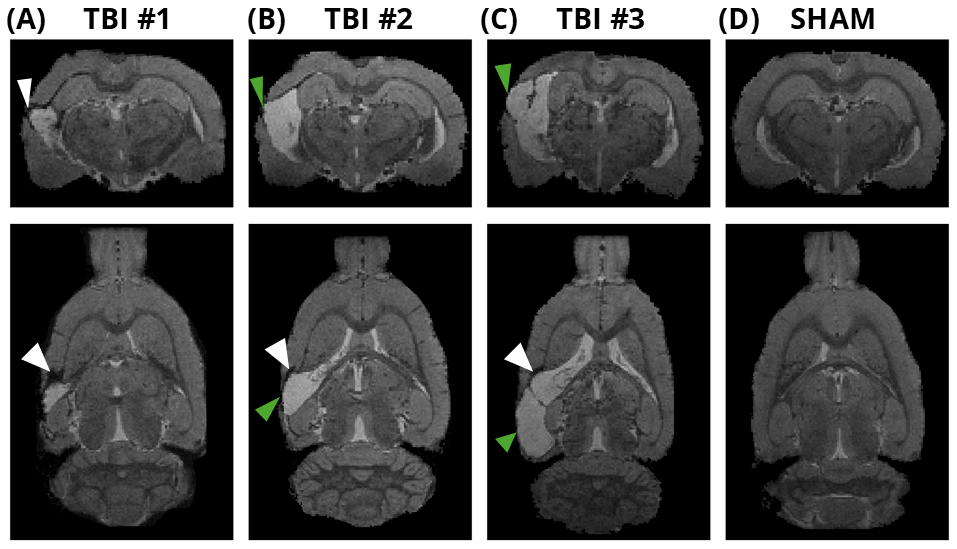


**Supplementary Figure 3**: Example of coronal and sagittal views of MGE anatomical images in three TBI (A-C) and one SHAM operated (D) animals at six months post-injury. Note the difference in the lesion severity and location. The white arrowhead denotes the signal hypointensity probably caused by microbleeds or iron residues, and the green arrowhead denotes cortical thinning.


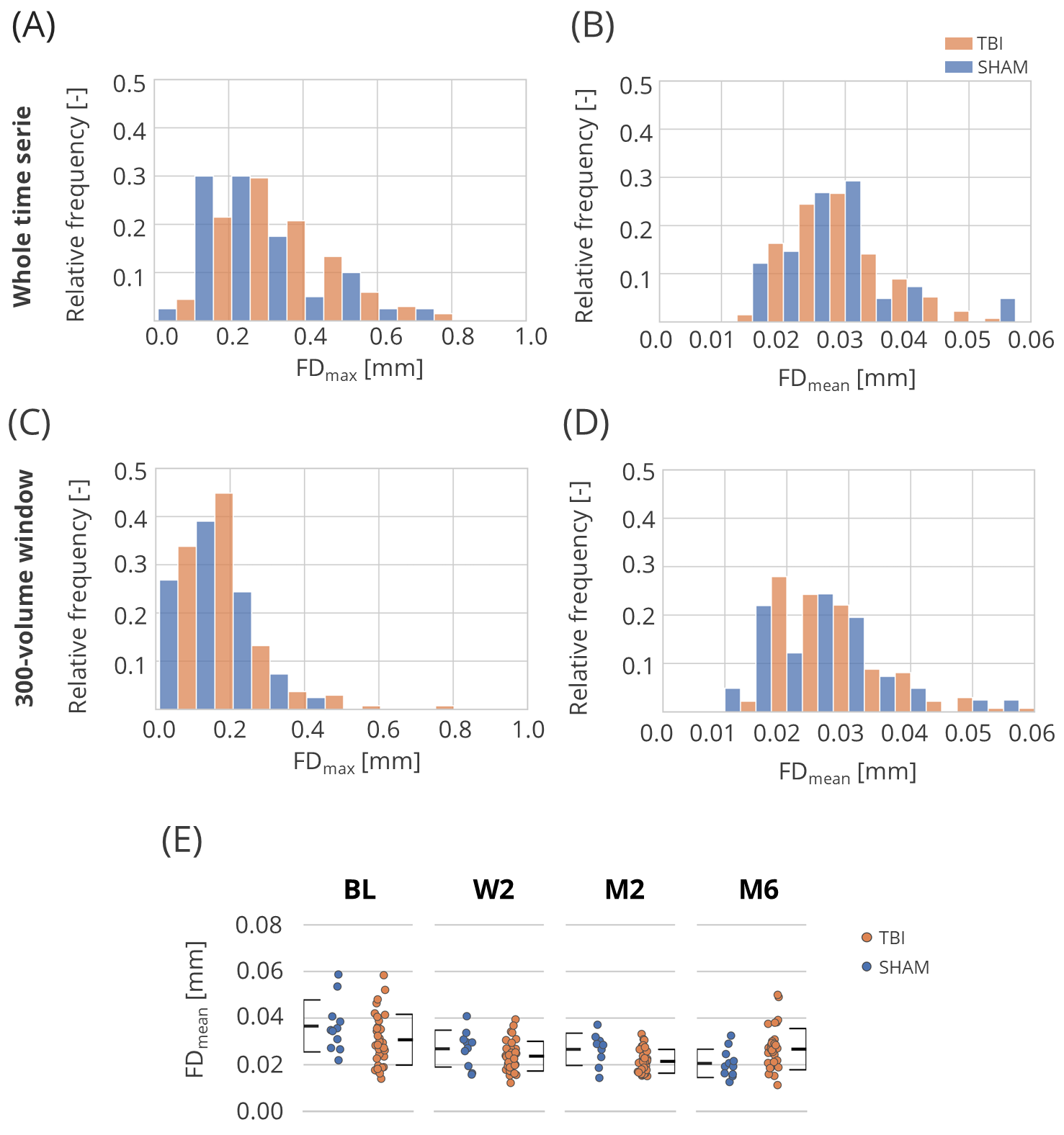


**Supplementary Figure 4**: Framewise displacement (FD) of the fMRI time series. (A-D) Relative frequency histograms. We estimated the maximum (A, C) and mean (B, D) framewise coefficients. They were calculated across the whole time-series (A, B) as well as within the 300-volume window used for the functional connectivity (FC) analysis (C, D). Overall, the FD was small, and the distributions were similar between TBI and SHAM animals. (E) The mean FD at each timepoint, no statistically significant differences were found between groups (two-sample t-test, p > 0.05). Error bars show the standard deviation.


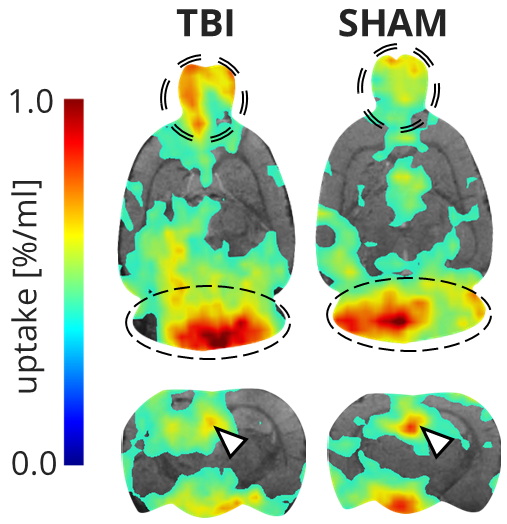


**Supplementary Figure 5**: The uptake of [^18^F]-FEPPA in representative TBI (right) and SHAM (middle) animals. The physiological uptake of this translocator protein (TSPO) tracer can be visible around vessels (white arrowhead), in the cerebellum (dashed line; --), and in the olfactory bulbs (dashed double line, ==) in both TBI and SHAM animals.


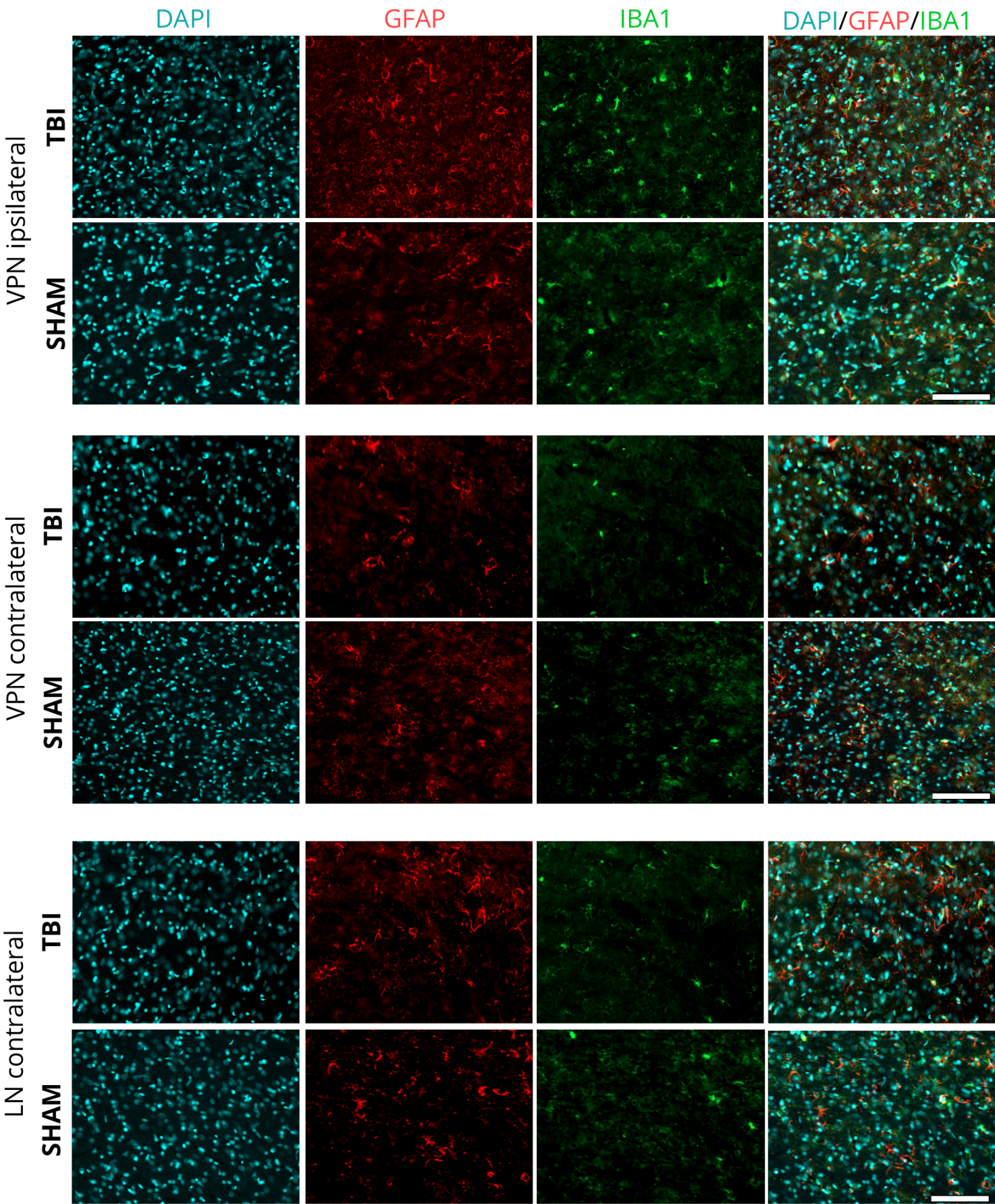


**Supplementary Figure 6:** Representative section of ipsilateral and contralateral ventral lateral nuclei (VPN) and contralateral lateral nuclei (LN) labeled with DAPI, GFAP, and IBA1 in TBI and SHAM animals. TBI animals show higher cellularity, GFAP, and IBA1 immunoreactivity in the ipsilateral VPN. Scale bar:100 µm

*
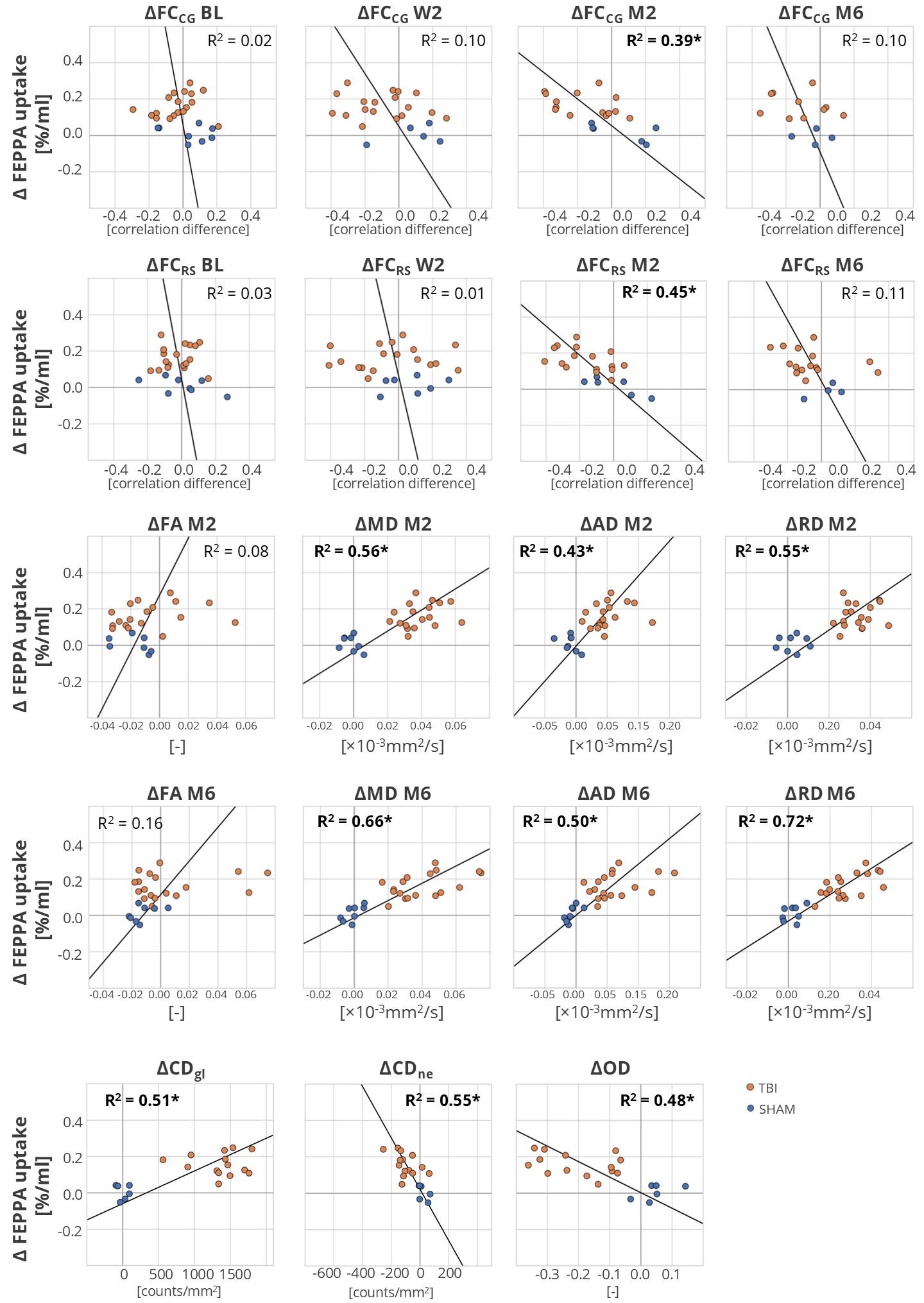
*

**Supplementary Figure 7:** Scatter plots of the ΔU_FEPPA_ in LN and corresponding differences of in other imaging and histological metrics and their respective linear regression line. Asterisk (*) denotes statistically significant linear regressions (q < 0.05). AD, axial diffusivity; BL, baseline; CD_gl_, glial cell density; CD_ne_, neuronal cell density; FA, fractional anisotropy; FC_CG_, thalamic functional connectivity to cingulate cortical area; FC_RS_, thalamic functional connectivity to retrosplenial cortical area; LN, lateral nuclei; MD, mean diffusivity; n.s.; non-significant; M2, 2 months post-injury; M6, six months post-injury; OD, optical density; RD, radial diffusivity; U_FEPPA_, uptake of the [^18^F]-FEPPA; W2, 2 weeks post-injury.

*
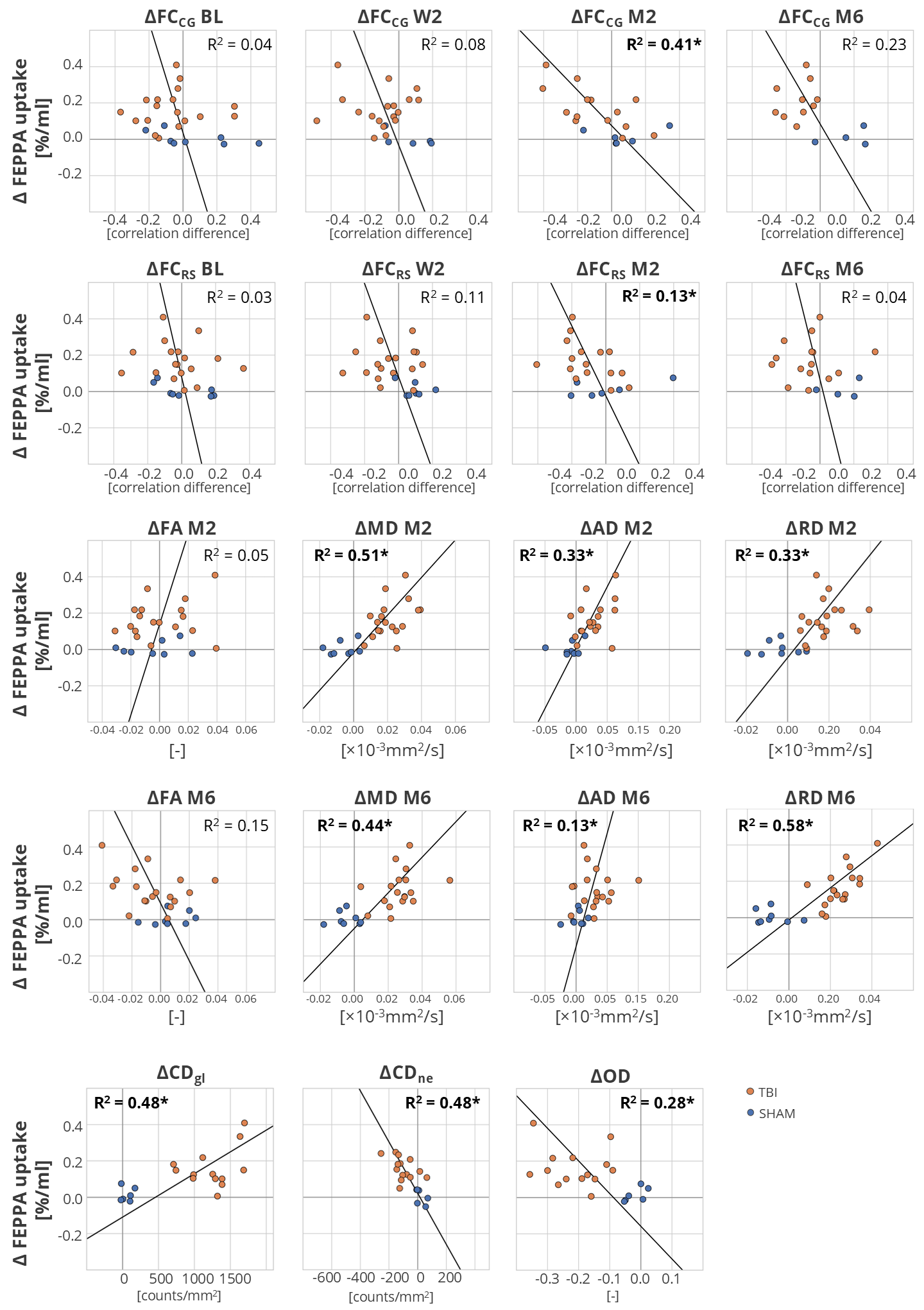
*

**Supplementary Figure 8:** Scatter plots of the ΔU_FEPPA_ in VPN and corresponding differences of in other imaging and histological metrics and their respective linear regression line. Asterisk (*) denotes statistically significant linear regressions (q < 0.05). AD, axial diffusivity; BL, baseline; CD_gl_, glial cell density; CD_ne_, neuronal cell density; FA, fractional anisotropy; FC_CG_, thalamic functional connectivity to cingulate cortical area; FC_RS_, thalamic functional connectivity to retrosplenial cortical area; MD, mean diffusivity; n.s.; non-significant; M2, 2 months post-injury; M6, six months post-injury; OD, optical density; RD, radial diffusivity; U_FEPPA_, uptake of the [F^18^]-FEPPA; VPN, ventral posterior nucleus; W2, 2 weeks post-injury.
