# Supplementary material for: Secondary thalamic neuroinflammation associates with disturbed corticothalamic connectivity in a model of severe traumatic brain injury in male rats—a longitudinal study
Source: Cereb Cortex. 2026 Jan 9;36(1):bhaf337. doi: 10.1093/cercor/bhaf337 (PMC12784941; doi:10.1093/cercor/bhaf337)
Supplement: Supplementary_Methods [file supplementary_methods.docx]

**Supplementary Methods**

Secondary thalamic neuroinflammation associates with disturbed corticothalamic connectivity in a model of severe traumatic brain injury in male rats – a longitudinal study

Lenka Dvořáková^1^, Raimo A. Salo^1^, Petteri Stenroos^1^, Kimmo Jokivarsi^1^, Jenni Kyyriäinen^1^, Ekaterina Paasonen^1,2^, Eppu Manninen^1^, Mikko Kettunen^1^, Pekka Poutiainen^3^, Alejandra Sierra^1^, Jaakko Paasonen^1^ and Olli Gröhn^1^*

*^1^A. I. Virtanen Institute for Molecular Sciences,* *University of Eastern Finland, Kuopio, Finland*

*^2^Neurocenter*, *Kuopio University Hospital, Kuopio, Finland*

*^3^Diagnostic Imaging Center, Kuopio University Hospital, Kuopio, Finland*

*Corresponding Author:

Professor Olli Gröhn, Ph.D.

e-mail: [olli.grohn@uef.fi](mailto:olli.grohn@uef.fi)

telephone: +358 50 3590963

mailing address:
A.I.V. Institute for Molecular Sciences, University of Eastern Finland
P.O. Box 1627
Neulaniementie 2,
FI-70211, Kuopio
Finland

**Tissue preparation and histology**

All animals were perfused, and the brains were prepared for histology. Rats were deeply anaesthetized with isoflurane before the transcardial perfusion with 0.9 % saline for 5 min and 4% paraformaldehyde (PFA) in phosphate-buffer saline for 20 min. Animals were subsequently decapitated and the brains extracted from the skull and post-fixed for 4 h in 4 % PFA. After the fixation, all the brains were immersed in a cryoprotective solution containing 20% glycerol in 0.02 M potassium phosphate-buffered saline (pH = 7.4) for 36 h. Then, the brains were blocked, frozen in dry ice, and preserved at –70 °C until sectioning.

The brains were sectioned in the coronal plane (30 μm, 1-in-5 series) using a sliding microtome. Sections from the first series were stored in 10% formalin in room temperature while the remaining series were stored in a cryoprotectant tissue-collecting solution (30% ethylene glycol, 25% glycerol in 0.05 M sodium phosphate buffer) at –20 °C until further processing.

The first series of sections was stained with thionin (Nissl) to assess the cytoarchitecture, gliosis, and severity of tissue damage after TBI. The second series of sections was stained with a gold chloride solution for myelin. Myelin staining was performed on sections mounted onto gelatin-coated slides and dried at 37˚C. The sections were then incubated in the dark for a period between 4-6 h in a solution containing gold chloride (HAuCl_4_·3H_2_O; G-4022, Sigma-Aldrich, MO, USA) in 0.02 M sodium phosphate buffer in 0.09% NaCl, pH 7.4. The slides were washed in 0.02 M sodium phosphate buffer in 0.09% NaCl twice for 4 min and placed in a 2.5% sodium thiosulfate solution for 5 min. After the procedure, the sections were washed three times in phosphate buffer for 10 min each. Finally, the sections were dehydrated through an ascending series of ethanol, cleared in xylene, and cover-slipped with DePeX mounting medium (BDH, Laboratory Supplies, Dorset, UK).

**Immunochemistry staining**

To investigate glial cell activation, sections were double labeled with Ionized calcium binding adaptor molecule 1 (IBA1) and Glial fibrillary acidic protein (GFAP). The following primary antibodies were used: polyclonal rabbit anti-rat Iba1 (1:250, 019-19741, Fujifilm Wako Pure Chemical Corporation, Japan) and polyclonal rabbit anti-GFAP (1:500, Z0334, Agilent Dako, Santa Clara, CA, USA). As secondary antibodies we used: goat anti-rabbit Alexa Fluor® 488 (1:300, A-11094, Invitrogen™, Thermo Fisher Scientific, Waltham, MA, USA), goat anti-rabbit Alexa Fluor® 647 (1:300, ab150079, Abcam, Cambridge, UK). The sections were rinsed three times in 0.1 M phosphate buffer (PB) (10 min each) and mounted on gelatin-coated slides. After air-drying, sections were rehydrated for 5 min in a solution containing phosphate buffered saline (PBS) and 0.05% TWEEN® 20 (T). Non-specific binding was blocked with solution containing 10% normal goat serum (NGS) in PBS-T. The sections were then incubated in a solution of anti-Iba1 in 5% NGS in PBS-T (overnight, 4°C), washed, and incubated with secondary antibody Alexa Fluor® 488 for 2 h. For double labeling, sections were washed in PBS-T (3 times, 5 min each) and incubated overnight (4°C) in a solution containing anti-GFAP in 5% NGS in PBS-T. After washing with PBS-T (3 times, 5 min each), the sections were incubated in secondary antibody Alexa Fluor® 647 for 2 h. Finally, the sections were washed, air-dried, and cover slipped with VECTASHIELD® Antifade Mounting Medium with DAPI (H-1200-10, Vector Laboratories, Newark, CA, USA). The cover glass was fastened with nail polish.
