# Supplementary material for: Secondary thalamic neuroinflammation associates with disturbed corticothalamic connectivity in a model of severe traumatic brain injury in male rats—a longitudinal study
Source: Cereb Cortex. 2026 Jan 9;36(1):bhaf337. doi: 10.1093/cercor/bhaf337 (PMC12784941; doi:10.1093/cercor/bhaf337)
Supplement: Supplementary_Tables_(1)_bhaf337 [file supplementary_tables_(1)_bhaf337.docx]

**Supplementary Tables**

Secondary thalamic neuroinflammation associates with disturbed corticothalamic connectivity in a model of severe traumatic brain injury in male rats – a longitudinal study

Lenka Dvořáková^1^, Raimo A. Salo^1^, Petteri Stenroos^1^, Kimmo Jokivarsi^1^, Jenni Kyyriäinen^1^, Ekaterina Paasonen^1,2^, Eppu Manninen^1^, Mikko Kettunen^1^, Pekka Poutiainen^3^, Alejandra Sierra^1^, Jaakko Paasonen^1^ and Olli Gröhn^1^*

*^1^A. I. Virtanen Institute for Molecular Sciences,* *University of Eastern Finland, Kuopio, Finland*

*^2^Neurocenter*, *Kuopio University Hospital, Kuopio, Finland*

*^3^Diagnostic Imaging Center, Kuopio University Hospital, Kuopio, Finland*

*Corresponding Author:

Professor Olli Gröhn, Ph.D.

e-mail: [olli.grohn@uef.fi](mailto:olli.grohn@uef.fi)

telephone: +358 50 3590963

mailing address:
A.I.V. Institute for Molecular Sciences, University of Eastern Finland
P.O. Box 1627
Neulaniementie 2,
FI-70211, Kuopio
Finland

**Supplementary Table 1:** Lateral asymmetry analysis of the functional connectivity (FC) of corticothalamic connections. Values shown as mean ± standard deviation. Statistical significance: * indicates q < 0.05 between ipsilateral and contralateral FC in a false discovery rate-corrected t-test. BL, baseline; FC_CG_, correlation coefficient of the thalamic functional connectivity to cingulate cortical area; FC_RS_, correlation coefficient of thalamic functional connectivity to retrosplenial cortical area; LN, lateral nuclei; M2, 2 months post-injury; M6, six months post-injury; VPN, ventral posterior nucleus, W2, 2 weeks post-injury.

|  |  |  | **FC_CG_** | |  |  | **FC_RS_** | |  |
| --- | --- | --- | --- | --- | --- | --- | --- | --- | --- |
|  |  |  | ipsilateral | contralateral |  |  | ipsilateral | contralateral |  |
| **TBI** | BL | LN | 0.59 ± 0.27 | 0.58 ± 0.30 |  |  | 0.50 ± 0.25 | 0.50 ± 0.26 |  |
|  |  | VPN | 0.41 ± 0.24 | 0.44 ± 0.24 |  |  | 0.34 ± 0.24 | 0.33 ± 0.18 |  |
|  | W2 | LN | **0.42 ± 0.21** | **0.53 ± 0.28*** |  |  | **0.39 ± 0.24** | **0.47 ± 0.29*** |  |
|  |  | VPN | 0.32 ± 0.20 | 0.37 ± 0.19 |  |  | 0.29 ± 0.21 | 0.33 ± 0.18 |  |
|  | M2 | LN | **0.45 ± 0.22** | **0.57 ± 0.27*** |  |  | **0.40 ± 0.26** | **0.52 ± 0.26*** |  |
|  |  | VPN | **0.36 ± 0.18** | **0.43 ± 0.21*** |  |  | 0.33 ± 0.19 | 0.39 ± 0.25 |  |
|  | M6 | LN | **0.37 ± 0.30** | **0.48 ± 0.34*** |  |  | 0.33 ± 0.32 | 0.38 ± 0.31 |  |
|  |  | VPN | **0.31 ± 0.28** | **0.40 ± 0.29*** |  |  | 0.27 ± 0.25 | 0.33 ± 0.26 |  |
| **SHAM** | BL | LN | 0.60 ± 0.30 | 0.57 ± 0.31 |  |  | 0.53 ± 0.30 | 0.52 ± 0.31 |  |
|  |  | VPN | 0.50 ± 0.25 | 0.48 ± 0.24 |  |  | 0.40 ± 0.25 | 0.41 ± 0.25 |  |
|  | W2 | LN | 0.65 ± 0.28 | 0.62 ± 0.26 |  |  | 0.55 ± 0.24 | 0.51 ± 0.29 |  |
|  |  | VPN | 0.50 ± 0.30 | 0.50 ± 0.16 |  |  | 0.39 ± 0.23 | 0.36 ± 0.23 |  |
|  | M2 | LN | 0.54 ± 0.34 | 0.56 ± 0.32 |  |  | 0.45 ± 0.21 | 0.46 ± 0.19 |  |
|  |  | VPN | 0.48 ± 0.43 | 0.48 ± 0.38 |  |  | 0.37 ± 0.39 | 0.39 ± 0.32 |  |
|  | M6 | LN | 0.59 ± 0.22 | 0.58 ± 0.26 |  |  | 0.48 ± 0.23 | 0.45 ± 0.23 |  |
|  |  | VPN | 0.47 ± 0.21 | 0.34 ± 0.23 |  |  | 0.29 ± 0.29 | 0.35 ± 0.24 |  |

**Supplementary Table 2:** Lateral asymmetry analysis of the [18F]-FEPPA uptake in the thalamic areas. Values shown as mean ± standard deviation. Statistical significance: * indicates q < 0.05 between ipsilateral and contralateral FC in a false discovery rate-corrected t-test. LN, lateral nuclei; VPN, ventral posterior nucleus, W2, 2 weeks post-injury.

|  |  |  | **U_FEPPA_ [%/ml]** | |  |
| --- | --- | --- | --- | --- | --- |
|  |  |  | ipsilateral | contralateral |  |
| **TBI** | W2 | LN | **0.77 ± 0.15** | **0.60 ± 0.11*** |  |
|  |  | VPN | **0.66 ± 0.14** | **0.49 ± 0.10*** |  |
| **SHAM** | W2 | LN | 0.56 ± 0.05 | 0.55 ± 0.06 |  |
|  |  | VPN | 0.46 ± 0.06 | 0.46 ± 0.06 |  |

**Supplementary Table 3:** Lateral asymmetry analysis of the DTI-derived metrics of thalamic and striatal ROIs. Values shown as mean ± standard deviation. Statistical significance: * indicates q < 0.05 between ipsilateral and contralateral FC in a false discovery rate-corrected t-test. AD, axial diffusivity; CG, cingulate cortical area; DMS, dorsal medial striatum; FA, fractional anisotropy; LN, lateral nuclei; M2, 2 months post-injury; M6, six months post-injury; MD, mean diffusivity; RD, radial diffusivity; VPN, ventral posterior nucleus.

|  |  |  | **MD [×10^-3^mm^2^/s]** | |  |  | **AD [×10^-3^mm^2^/s]** | |  |  | **RD [×10^-3^mm^2^/s]** | |  |  | **FA [-]** | |
| --- | --- | --- | --- | --- | --- | --- | --- | --- | --- | --- | --- | --- | --- | --- | --- | --- |
|  |  |  | ipsilateral | contralateral |  |  | ipsilateral | contralateral |  |  | ipsilateral | contralateral |  |  | ipsilateral | contralateral |
| **TBI** | M2 | LN | **0.62 ± 0.02** | **0.58 ± 0.02*** |  |  | **0.73 ± 0.03** | **0.69 ± 0.03*** |  |  | **0.56 ± 0.02** | **0.52 ± 0.02*** |  |  | **0.18 ± 0.02*** | **0.20 ± 0.02*** |
|  |  | VPN | **0.57 ± 0.02** | **0.55 ± 0.02*** |  |  | 0.72 ± 0.03 | 0.71 ± 0.03 |  |  | **0.50 ± 0.02** | **0.47 ± 0.02*** |  |  | 0.25 ± 0.02 | 0.26 ± 0.02 |
|  |  | DMS | 0.59 ± 0.02 | 0.59 ± 0.02 |  |  | 0.80 ± 0.03 | 0.78 ± 0.03 |  |  | 0.49 ± 0.02 | 0.49 ± 0.02 |  |  | 0.31 ± 0.01 | 0.30 ± 0.03 |
|  | M6 | LN | **0.63 ± 0.03** | **0.58 ± 0.02*** |  |  | **0.76 ± 0.05** | **0.70 ± 0.02*** |  |  | **0.56 ± 0.02** | **0.53 ± 0.02*** |  |  | 0.19 ± 0.03 | 0.20 ± 0.02 |
|  |  | VPN | **0.59 ± 0.02** | **0.56 ± 0.02*** |  |  | **0.75 ± 0.03** | **0.72 ± 0.02*** |  |  | **0.51 ± 0.02** | **0.48 ± 0.02*** |  |  | 0.25 ± 0.02 | 0.26 ± 0.02 |
|  |  | DMS | 0.60 ± 0.02 | 0.60 ± 0.02 |  |  | 0.81 ± 0.03 | 0.80 ± 0.04 |  |  | 0.50 ± 0.02 | 0.49 ± 0.02 |  |  | 0.31 ± 0.03 | 0.31 ± 0.03 |
| **SHAM** | M2 | LN | 0.59 ± 0.02 | 0.59 ± 0.02 |  |  | 0.70 ± 0.03 | 0.71 ± 0.03 |  |  | 0.53 ± 0.02 | 0.53 ± 0.02 |  |  | 0.19 ± 0.02 | 0.20 ± 0.02 |
|  |  | VPN | 0.56 ± 0.02 | 0.57 ± 0.02 |  |  | 0.72 ± 0.03 | 0.73 ± 0.03 |  |  | 0.48 ± 0.02 | 0.48 ± 0.02 |  |  | 0.27 ± 0.02 | 0.27 ± 0.02 |
|  |  | DMS | 0.60 ± 0.02 | 0.59 ± 0.02 |  |  | 0.81 ± 0.02 | 0.80 ± 0.03 |  |  | 0.50 ± 0.02 | 0.48 ± 0.02 |  |  | 0.31 ± 0.02 | 0.31 ± 0.01 |
|  | M6 | LN | 0.59 ± 0.02 | 0.59 ± 0.02 |  |  | 0.71 ± 0.03 | 0.72 ± 0.03 |  |  | 0.53 ± 0.02 | 0.53 ± 0.02 |  |  | 0.19 ± 0.02 | 0.21 ± 0.01 |
|  |  | VPN | 0.56 ± 0.02 | 0.57 ± 0.02 |  |  | 0.73 ± 0.03 | 0.73 ± 0.03 |  |  | 0.47 ± 0.02 | 0.48 ± 0.01 |  |  | 0.28 ± 0.02 | 0.27 ± 0.02 |
|  |  | DMS | 0.60 ± 0.02 | 0.59 ± 0.01 |  |  | 0.81 ± 0.02 | 0.80 ± 0.02 |  |  | 0.50 ± 0.02 | 0.49 ± 0.02 |  |  | 0.30 ± 0.01 | 0.31 ± 0.02 |

**Supplementary Table 4:** Lateral asymmetry analysis of the histology-derived metrics of thalamic and striatal ROIs. Values shown as mean ± standard deviation. Statistical significance: * indicates q < 0.05 between ipsilateral and contralateral FC in a false discovery rate-corrected t-test. AD, axial diffusivity; CD_gl_, glial cell density; CD_ne_, neuronal cell density; CG, cingulate cortical area; FA, fractional anisotropy; LN, lateral nuclei; M8, eight months post-injury; MD, mean diffusivity; OD, optical density; RD, radial diffusivity; VPN, ventral posterior nucleus.

|  |  |  | **CD_gl_ [counts/mm^2^]** | |  |  | **CD_ne_ [counts/mm^2^]** | |  |  | **OD [-]** | |  |
| --- | --- | --- | --- | --- | --- | --- | --- | --- | --- | --- | --- | --- | --- |
|  |  |  | ipsilateral | contralateral |  |  | ipsilateral | contralateral |  |  | ipsilateral | contralateral |  |
| **TBI** | M8 | LN | **3395 ± 388** | **2037 ± 161*** |  |  | **879 ± 74** | **979 ± 75*** |  |  | **0.20 ± 0.05** | **0.31 ± 0.05*** |  |
|  |  | VPN | **3562 ± 591** | **2224 ± 205*** |  |  | **648 ± 117** | **882 ± 44*** |  |  | **0.42 ± 0.06** | **0.54 ± 0.06*** |  |
|  |  | RS | **2479 ± 190** | **2319 ± 159*** |  |  | **1283 ± 62** | **1236 ± 61*** |  |  | **0.19 ± 0.03** | **0.23 ± 0.04*** |  |
| **SHAM** | M8 | LN | 2003 ± 181 | 2000 ± 184 |  |  | 957 ± 44 | 936 ± 65 |  |  | 0.32 ± 0.05 | 0.30 ± 0.05 |  |
|  |  | VPN | 2251 ± 137 | 2193 ± 209 |  |  | 896 ± 49 | 870 ± 27 |  |  | 0.56 ± 0.03 | 0.56 ± 0.03 |  |
|  |  | RS | 2300 ± 284 | 2415 ± 257 |  |  | 1203 ± 68 | 1178 ± 87 |  |  | **0.20 ± 0.06** | **0.24 ± 0.04*** |  |
